# A conditional threat-regulation model: When deliberative processing of ongoing threat reduces intergroup bias

**DOI:** 10.1093/pnasnexus/pgag295

**Published:** 2026-09-01

**Authors:** Carmit T Tadmor, Melody M Chao, Ying-yi Hong, Lawrance Y Cai

**Affiliations:** Coller School of Management, Tel Aviv University, Tel Aviv, Israel; Department of Management, Hong Kong University of Science and Technology, Hong Kong SAR, China; Department of Psychology, The Chinese University of Hong Kong, Hong Kong SAR, China; Faculty of Business and Economics, University of Hong Kong, Hong Kong SAR, China

**Keywords:** expressive writing, deliberative processing, societal threat, intergroup bias, integrative complexity, multicultural experience

## Abstract

Ongoing societal threats such as war, civil unrest, and pandemics heighten intergroup bias by inducing cognitive rigidity and suppressing complex reasoning. Yet most bias-reduction strategies require conditions that threat itself undermines, including direct engagement with outgroups. We propose a conditional threat-regulation model that specifies when and for whom threat-induced cognitive rigidity can be regulated in the face of threat. Across three experiments conducted during real societal crises in Israel, Hong Kong SAR, and the United States, we test whether deliberative processing of ongoing threat can enable integrative complexity and thereby reduce intergroup bias toward groups associated with the threat. Two exploratory studies conducted during armed conflict and societal unrest varied writing topic (threat vs. nonthreat) while holding depth of processing constant; deliberative processing of the ongoing threat increased integrative complexity conditionally and in turn was associated with reduced intergroup bias. A preregistered experiment conducted during the COVID-19 pandemic crossed writing topic with depth of processing (deliberative vs. intuitive) to isolate the role of deliberation, and demonstrated that deliberative processing of ongoing threat enabled greater integrative complexity and reduced intergroup bias. Across studies, these effects were conditional on individuals’ cognitive preparedness, indexed by multicultural experience, with benefits concentrated among individuals able to translate deliberation into integratively complex reasoning. Integrative complexity served as a structural signature of regulated cognition under threat, mediating the relationship between deliberative processing and intergroup judgment. Together, the findings indicate that regulation of threat-induced cognitive rigidity is possible but conditional on both situational prompts and individual cognitive readiness.

Significance Statement Societal threats like war, civil unrest, and pandemics often intensify intergroup negativity, fueling conflict escalation. This research introduces the conditional threat-regulation model to identify a cognitive pathway through which intergroup bias may be reduced under threat, without requiring direct intergroup contact. Across three societal crises, findings suggest that deliberative processing may contribute to lower bias particularly toward groups associated with the threat through complex reasoning but only under specific conditions. Specifically, these effects depend on both situational prompts that encourage deliberation and individuals’ cognitive preparedness, indexed by multicultural experience. By revealing when and for whom threat-induced rigidity may be regulated, this work advances understanding of intergroup bias during societal crises and points toward targeted approaches to reducing bias under threat.

## Introduction

Ongoing societal threats—like wars, pandemics, or civil unrest—pose critical challenges to society by triggering defensive responses and derogation of groups associated with the threat (1–5). This occurs because threat induces narrowing of cognitive scope, biasing information processing and thereby fostering cognitive rigidity that underpins intergroup bias (6–12). In short, threat induces fast, intuitive “System 1” responses that simplify perception and amplify reliance on stereotypes and heuristic judgments. Accordingly, heightened intergroup bias under threat reflects a shift toward automatic, defensive processing. Most established bias-reduction strategies, such as intergroup contact (13–15), presume openness and engagement that are often compromised under ongoing societal threat. In an era of escalating global conflict and societal unrest (16), a central theoretical challenge is identifying psychological mechanisms that can counter threat-induced cognitive rigidity and reduce biased social judgment in the face of threat.

To address this challenge, we propose the conditional threat-regulation (CTR) model. The CTR model posits that threat-induced intergroup bias can be regulated by targeting the cognitive architecture of social judgment. Specifically, the model identifies a pathway to bias reduction conditioned on the interaction between deliberative processing interventions and an individual's cognitive preparedness for complex reasoning. It recognizes that ongoing threat increases reliance on intuitive system 1 processing, prompting rigid and oversimplified interpretations of social groups and increasing bias against outgroups. Reducing bias therefore depends on engaging deliberative system 2 processing. The model centers on how these processing dynamics can be regulated under threat, suggesting that the effectiveness of regulation is conditional on a situational trigger for deliberation and individual capacity for complex information processing.

When threat-induced cognitive rigidity is successfully regulated by engaging system 2, social judgment should no longer be dominated by simplified, biased interpretations. It could instead lead to greater differentiation among competing perspectives and a capacity to reconcile them in a coherent manner, hallmarks of integrative complexity. Integrative complexity captures the structure of thought, indexing the extent to which individuals recognize and integrate multiple, potentially conflicting viewpoints into a more nuanced understanding (10, 11, 17–19). Under ongoing threat, this form of reasoning often gives way to categorical and one-sided judgments that heighten negative evaluations of threat-associated outgroups (20). Interventions that successfully regulate threat-induced processing can increase integrative complexity and thereby create conditions for less biased social evaluations. Because cognitive rigidity under threat is organized around the threatening stimulus itself, changes in integrative complexity should matter most for judgments about groups associated with the ongoing threat, where defensive simplification is strongest. This logic is also consistent with prior work showing that threat produces negative attitudes toward groups associated with the threat rather than outgroups in general (21–24).

Importantly, the CTR model differentiates the depth of processing (deliberative vs. intuitive) from the resulting cognitive structure (low vs. high integratively complex thought). Deliberative processing refers to effortful “system 2” engagement, whereas intuitive processing refers to autonomous “system 1” responding. High and low integrative complexities describe the structure of thought, with high integrative complexity characterized by recognizing multiple facets of diverging perspectives and being able to reconcile and integrate apparently contradictory perspectives, and low integrative complexity characterized by rigid black-and-white thought. In the CTR framework, deliberation is a mechanism through which integratively complex reasoning can emerge.

The expressive-writing literature offers a useful way to engage deliberative processing and regulate threat-induced cognitive rigidity. Expressive writing is a well-established method for reflecting on emotionally charged experiences (25), with evidence demonstrating benefits across a wide range of outcomes, from improved well-being to fewer stress-related medical visits (25, 26). A meta-analytic review provides converging support for its effectiveness (27), and other research documents meaningful effects for writing tasks as brief as 2 min across 2 days (28) and single short sessions (29). Critically, expressive writing is not merely cathartic. It can promote cognitive complexity by facilitating construction of coherent narratives (25, 30, 31), dampening emotional intensity in ways that allow multiple perspectives to be considered (32, 33), and reducing intrusive thoughts and freeing working memory resources needed for effortful reasoning (34–36). By prompting individuals to deliberatively process their experience, expressive writing can serve as a catalyst for complex information processing that is otherwise suppressed under threat.

At the same time, expressive writing does not uniformly produce adaptive outcomes. Although it is widely regarded as an effective tool to process emotional stimuli, it can generate an immediate increase in negative affect as individuals confront distressing experiences (26). That is, when deliberating about the experience of threats, expressive writing may foster rumination (37). In such cases, elaborating on threatening experiences might reinforce existing biased reactions. Under ongoing societal threat, where emotionally charged material is already salient, this pattern may strengthen defensive modes of thought rather than weaken them.

From the perspective of the CTR model, the diverging effects of expressive writing highlight that engaging deliberation opens the possibility of bias reduction without guaranteeing it. Deliberative processing creates an opportunity for integratively complex reasoning, but its realization depends on individuals’ capacity to translate deliberation into complex thought. When that translation does not occur, expressive writing may amplify existing interpretations of threat, including rigid evaluations of outgroups associated with the threat. Accordingly, the effectiveness of deliberative interventions is conditional on individuals’ cognitive preparedness for integratively complex reasoning. Individuals differ in the extent to which they can capitalize on deliberative prompts. In particular, those with more multicultural experience tend to possess greater capacity to engage in integrative complex reasoning once deliberation is activated. Thus, the CTR model posits that multicultural experience is an important boundary condition shaping whether expressive writing translates into integratively complex thoughts and less biased social judgments.

Specifically, sustained engagement with multiple cultures requires individuals to navigate differing norms and reconcile ompeting values through differentiation and integration: recognizing the validity of multiple perspectives and combining them into more complex understandings. With repeated practice, such resolution can foster integrative complexity as an adaptive coping response that generalizes beyond the cultural domain into a readily accessible habit of information processing (12, 18, 20, 38). Evidence across correlational, experimental, and longitudinal studies demonstrates that multicultural experience can enhance integrative complexity, over and above intelligence, personality, and academic performance (39–43). While these readily accessible habits do not shield individuals from the cognitive narrowing effects of threat, they provide blueprints for integratively complex thoughts when deliberation is induced. Consequently, when engaging in expressive writing, individuals with greater multicultural experience are more likely to translate deliberative processing into integratively complex thoughts, leading to less biased social judgments than those with less multicultural experience.

Expressive writing therefore serves as a means to engage deliberative processing under threat, creating the conditions for integrative complexity. As individuals with high multicultural experience possess the cognitive habits to capitalize on deliberative processing, this results in a measurable integrative-complexity signature that leads to less biased social judgments especially toward groups associated with the threat.

**Figure 1:**
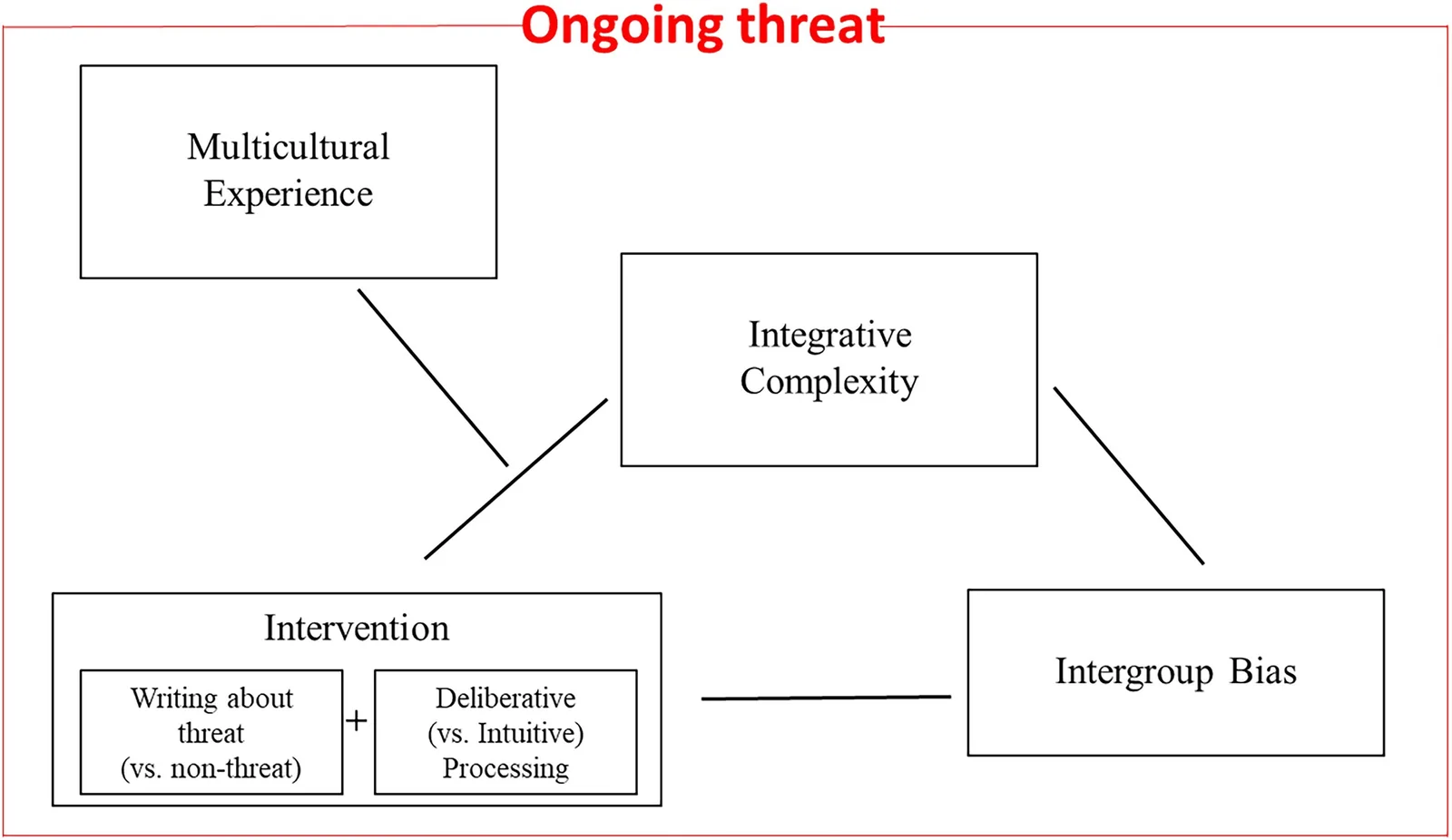
The CTR model. The model specifies the interactive effects of writing topic (threat vs. nonthreat), deliberative processing (vs. intuitive processing) and multicultural experience on integrative complexity, and the resulting effect on intergroup bias under ongoing societal threat.

We test the proposed CTR model (Fig. 1) in three experiments conducted during ongoing societal threats across distinct regions and conflict contexts. Experiments 1a and 1b vary writing topic (threat vs. nonthreat) while holding deliberative processing constant. Experiment 2 crosses writing topic with depth of processing (deliberative vs. intuitive), allowing us to isolate the role of deliberative processing under threat. Across studies, we examine whether deliberative processing of threat experiences elicits integrative complexity and whether integrative complexity predicts less biased judgments toward threat-associated groups, particularly among individuals higher in multicultural experience. Full methodological details are provided in the Supplementary material. Table S1a–c reports correlations among key variables for experiments 1a, 1b, and 2, respectively. Table S2a–c presents full bootstrapped moderated mediation results corresponding to the primary models tested in each experiment. Table S2d and e reports the sensitivity analyses excluding influential observations. All anonymized aggregated data and analytic syntax supporting the findings of the studies are publicly available via the Open Science Framework (OSF) repository at https://osf.io/6pb2s/overview?view_only=da3dbb48c3dc4fed9e536f9c088ba11a.

## Experiments 1a and 1b

As an exploratory test of the CTR model, we conducted two experiments during periods of ongoing societal threat. Experiment 1a was conducted in Israel during August 2014, amid military escalation and sustained missile attacks. Experiment 1b was conducted during November 2014 in Hong Kong SAR, a period of societal unrest following the announcement of proposed electoral reforms.

### Methods

#### Participants

In experiment 1a, the final sample consisted of 75 Jewish-Israeli students (53.3% women; *M*_age_ = 26.17, SD = 4.94). In experiment 1b, the final sample consisted of 124 Hong Kong Chinese undergraduates (50.8% women; *M*_age_ = 19.07, SD = 0.70). Given the time-sensitive nature of the crises, sample sizes were necessarily constrained by the recruitment windows available while the events were active, with duration that could not be anticipated. All participants provided informed consent before taking part in the experiments, and the experiments were approved, respectively, by the ethics review board of Tel Aviv University and the Committee on Research Practices of the Hong Kong University of Science and Technology (Protocol #90).

#### Materials and procedure

In both experiments, participants completed two ostensibly unrelated studies within a single online session. In the first study, all participants received deliberative processing instructions and writing topic was randomly assigned. Specifically, they were asked to deeply reflect on their thoughts and feelings about either the ongoing societal threat (armed conflict in experiment 1a; societal unrest in experiment 1b) or on a nonthreat topic (dental visit). Both experiments were conducted during ongoing real-world crises; thus, this manipulation varied whether participants explicitly reflected on the focal threat while holding depth of processing constant. This allowed us to test whether the predicted effects were specific to reflecting on the ongoing threat rather than an artifact of deliberative processing in general. The dentist prompt was chosen as a control based on existing work (44–47), because it allowed us to distinguish effects of the focal threat from effects of general unpleasantness. Essays were coded for topic engagement as a manipulation check to ensure that participants engaged with the assigned topic as instructed; full coding rules and descriptive results are reported in the Supplementary material. In the second ostensibly unrelated study, participants completed measures assessing intergroup attitudes toward groups associated with the focal threat. Participants then reported demographic information, including multicultural experience, and were debriefed.

#### Measures

Integrative complexity was assessed by scoring participants’ writing using standard procedures (17, 48). Integrative complexity captured information processing and is assessed by the structure of thought, not its content. Thus, the specific content of the writing (e.g. threat or dental experience) does not drive the integrative complexity score; rather, the structural complexity of the reasoning expressed in the essay does. The coding entails an assessment of the extent to which differentiation and integration are present in the text. Evidence for differentiation consists of references to more than one dimension of a problem or more than one perspective on an issue. Integration exists when differentiated elements are linked conceptually. The lowest score reflects a single, undifferentiated perspective; higher scores reflect greater differentiation among perspectives, and the highest scores reflect integration of differentiated perspectives. Intergroup bias was measured using established scales assessing negative evaluations of the outgroup perceived to be associated with the ongoing societal threat in each context (Palestinians in experiment 1a; Mainland Chinese in experiment 1b (20, 49–51). Multicultural experience was assessed using the Multicultural Experience Survey (12, 52).

### Results

We first tested the mediation effect of integrative complexity. Across both experiments, writing about the threat (vs. nonthreat) topic predicted higher integrative complexity (experiment 1a: *b* = 0.28, *P* = 0.02, 95% CI [0.05–0.51]; experiment 1b: *b* = 0.25, *P* = 0.01, 95% CI [0.08–0.42]) and higher integrative complexity was associated with lower intergroup bias (experiment 1a: *b* = −0.54, *P* < 0.001, 95% CI [−0.79, −0.28]; experiment 1b: *b* = −0.27, *P* < 0.001, 95% CI [−0.39, −0.15]). Bootstrapped mediation analyses (5,000 resamples) showed a significant indirect effect of the writing-topic manipulation on intergroup bias via integrative complexity, with higher integrative complexity associated with lower intergroup bias (experiment 1a: *b* = −0.12, 95% CI [−0.25, −0.02]; experiment 1b (*b* = −0.10, 95% CI [−0.19, −0.03]).

We next tested whether multicultural experience (mean-centered) moderated the effect of the writing-topic manipulation on integrative complexity. Our results showed significant main effects of writing about threat (vs. nonthreat) on integrative complexity (experiment 1a: *b* = 0.28, *P* = 0.01, 95% CI [0.07–0.50]; experiment 1b: *b* = 0.24, *P* = 0.003, 95% CI [0.09–0.40]) and of multicultural experience (experiment 1a: *b* = 0.44, *P* = 0.002, 95% CI [0.16–0.73]; experiment 1b: *b* = 0.68, *P* < 0.001, 95% CI [0.43–0.93]). These were qualified by the predicted writing topic × multicultural experience interaction in both experiments (experiment 1a: *b* = 0.33, *P* = 0.02, 95% CI [0.04–0.61]; experiment 1b: *b* = 0.37, *P* = 0.004, 95% CI [0.12–0.63]).

Follow-up simple slope analyses showed that writing about the threat (vs. nonthreat) was associated with greater integrative complexity only among participants high (+1 SD) in multicultural experience (experiment 1a: *b* = 0.55, *P* = 0.001, 95% CI [0.24–0.87]; experiment 1b: *b* = 0.49, *P* < 0.001, 95% CI [0.26–0.71]). No association emerged among participants low (−1 SD) in multicultural experience (experiment 1a: *b* = 0.02, *P* = 0.92, 95% CI [−0.30, 0.33]; experiment 1b: *b* = −0.002, *P* = 0.99, 95% CI [−0.23, 0.23]).

**Figure 2:**
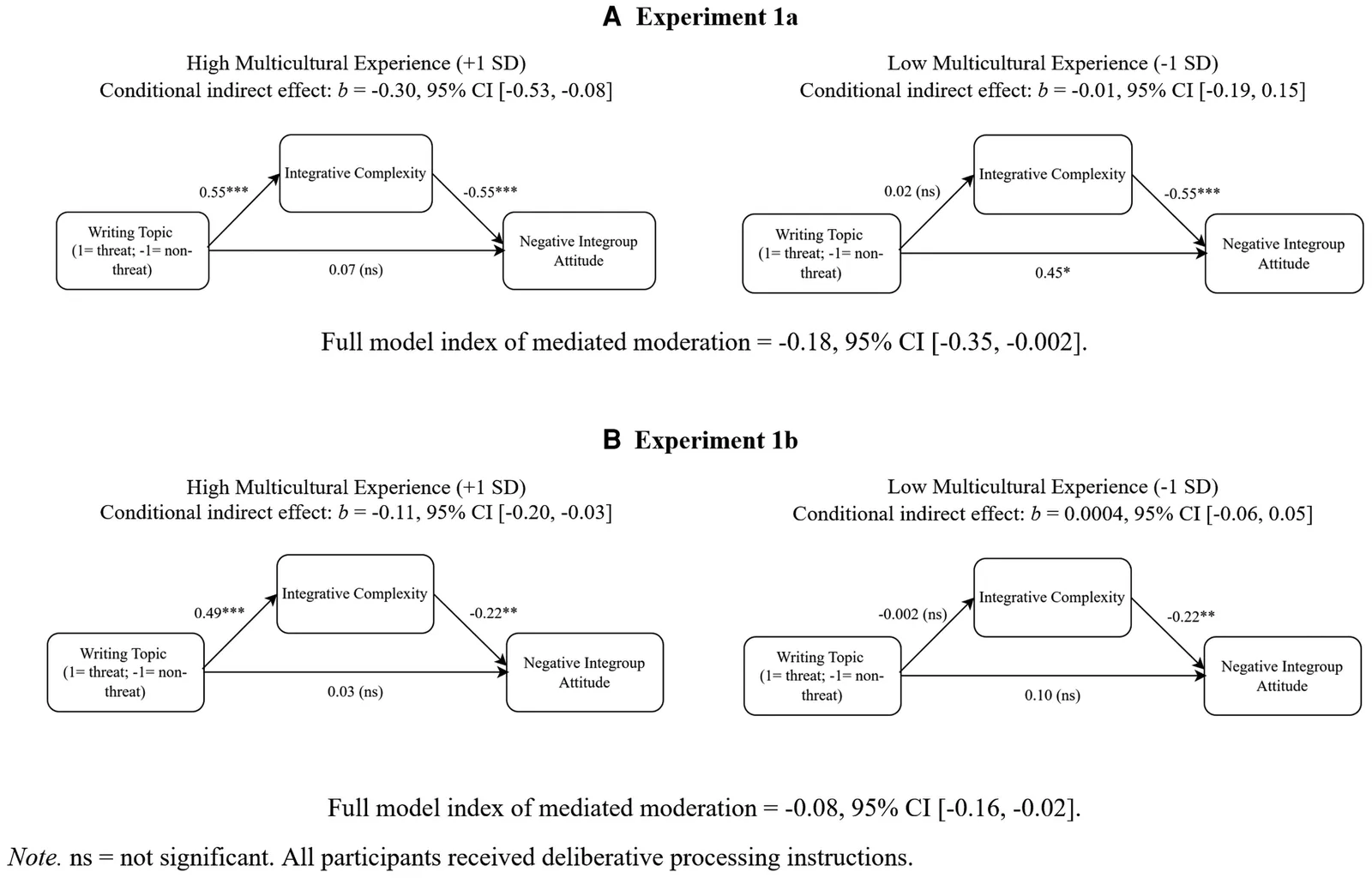
The conditional indirect effect of writing topic (threat vs. nonthreat) under deliberative processing on intergroup bias through integrative complexity, at high (+1 SD) and low (−1 SD) levels of multicultural experience, for (a) experiment 1a and (b) experiment 1b.

We next tested whether the indirect effect of the writing-topic manipulation on intergroup bias via complexity was conditional on multicultural experience. As shown in Fig. 2, among participants high (+1 SD) in multicultural experience, the indirect effect was significant (experiment 1a: *b* = −0.30, 95% CI [−0.53, −0.08]; experiment 1b: *b* = −0.11, 95% CI [−0.20, −0.03]). No significant indirect effect emerged among participants low (−1 SD) in multicultural experience (experiment 1a: *b* = −0.01, 95% CI [−0.19, 0.15]; experiment 1b: *b* = 0.004, 95% CI [−0.06, 0.05]). The indices of mediated moderation were significant in both experiments (experiment 1a: *b* = −0.18, 95% CI [−0.35, −0.002]; experiment 1b: *b* = −0.08, 95% CI [−0.16, −0.02]).

Although the CTR model predicts effects of writing-topic manipulation on intergroup bias through integrative complexity conditional on multicultural experience, we also examined the direct writing topic × multicultural experience interaction effect on intergroup bias for completeness. This interaction was significant in experiment 1a (*b* = −0.41, *P* = 0.03, 95% CI [−0.76, −0.05]) but not in experiment 1b (*b* = −0.13, *P* = 0.17, 95% CI [−0.33, 0.06]). In experiment 1a, writing about ongoing threat predicted greater intergroup bias among participants low (−1 SD) in multicultural experience (*b* = 0.44, *P* = 0.03, 95% CI [0.04–0.84]), but was nonsignificant among participants high (+1 SD) in multicultural experience (*b* = −0.23, *P* = 0.26, 95% CI [−0.63, 0.17]).

## Experiment 2

Experiments 1a and 1b held depth of processing constant as all participants received deliberative processing instructions and only the writing topic varied. These experiments demonstrated that deliberative processing of threat experiences could contribute to bias reduction, particularly for individuals with the cognitive preparedness to translate deliberation into integratively complex reasoning, whereas deliberative processing of an unrelated topic did not show such effects. Next, as a mechanism test of the CTR model, experiment 2 examined whether the benefits of writing about ongoing threat depended on deliberative processing, or whether engaging the threat topic without deliberation was sufficient. Within the CTR framework, deliberative processing of ongoing threat is central to whether threat-induced cognitive rigidity gives way to integratively complex reasoning. To examine the role of deliberative processing, experiment 2 crossed writing topic (threat vs. nonthreat) with depth of processing (deliberative vs. intuitive), with multicultural experience measured as a continuous moderator. Drawing on dual-process research showing that deliberative processing can be inhibited by cognitive load (53, 54), we predicted that deliberative (vs. intuitive) processing of ongoing threat would increase integrative complexity, which in turn was associated with reduced bias toward groups associated with the threat. Critically, this effect would be found among participants with the cognitive preparedness, indexed by multicultural experience, to translate deliberation into integratively complex reasoning. This preregistered experiment (https://aspredicted.org/blind.php?x=sh9zw8) was conducted during the COVID-19 pandemic in the United States to examine the generalizability of the model across threat contexts and populations.

### Methods

#### Participants

Participants were White American adults recruited via Prolific in May 2020 and compensated for participation. The final sample consisted of 415 participants (52.5% men; *M*_age_ = 34.29, SD = 12.25). Sample size was planned based on the observed effect sizes from experiments 1a and 1b. All participants provided informed consent before taking part in the experiments, and the experiments were approved by the Committee on Research Practices of the Hong Kong University of Science and Technology.

#### Materials and procedure

The study employed a 2 (writing topic: threat vs. nonthreat) × 2 (depth of processing: deliberative vs. intuitive) between-subjects design, with multicultural experience measured as a continuous moderator. This study was conducted in the United States during the height of the COVID-19 pandemic, a period marked by heightened social anxiety and polarization. Public discourse surrounding the origins of the virus coincided with intensified prejudice particularly toward Asian communities, alongside stigmatization and disproportionate impact of the pandemic experienced by Black communities (see Supplementary material for documentation of these threat associations). As in experiments 1a and 1b, the focal threat was present in the background of participants’ everyday lives. For the writing-topic manipulation, participants were randomly assigned to write about either their experiences related to the COVID-19 pandemic or a nonthreat topic (last movie/TV program watched). Using this nonthreat comparison in the experiment allowed us to further test whether the predicted effects depended on engaging the focal threat experiences regardless of the affective tone of the nonthreat topic. Our approach is consistent with the meta-analytic evidence that the effect of reflecting on threat experience is not driven by control-topic valence (44). As in experiments 1a and 1b, the essays were coded for topic engagement as a manipulation check; full descriptive results are reported in the Supplementary material.

Depth of processing was manipulated using established dual-process procedures. In the deliberative processing condition, participants were instructed to deeply reflect on their thoughts and feelings about the assigned topic. In the intuitive condition, participants were asked to memorize a dot pattern. While holding that pattern in mind, they wrote the first response that came to mind about their assigned topic. The dot-memorization task occupies the executive resources required for deliberation, and the “first response” instruction directed the respondents to engage in intuitive thinking (53–55). Together, these manipulations provide a robust induction of intuitive processing during the writing task. Thus, the design allowed us to test the CTR model's central prediction: that the effect of engaging the ongoing threat in writing on integrative complexity depends on whether participants have the deliberative resources to process it.

Integrative complexity was coded from participants’ essays; multicultural experience was assessed following the writing task. As dependent variables, we assessed prejudice toward Asian and Black targets.

### Results

We first tested the moderating effect of depth of processing. We found that writing about the threat (vs. nonthreat) predicted higher integrative complexity (*b* = 0.21, *P* < 0.001, 95% CI [0.15–0.27]), as did deliberative (vs. intuitive) processing (*b* = 0.24, *P* < 0.001, 95% CI [0.18–0.30]), with these effects qualified by the writing topic × depth of processing interaction (*b* = 0.08, *P* = 0.01, 95% CI [0.02–0.14]). Simple slope analyses indicated that writing about the threat (vs. nonthreat) increased integrative complexity under both processing conditions, but more strongly under deliberative processing (*b* = 0.29, *P* < 0.001, 95% CI [0.20–0.37]) than under intuitive processing (*b* = 0.13, *P* = 0.001, 95% CI [0.05–0.21]).

Higher integrative complexity predicted lower stereotype endorsement toward both Asian (*b* = −0.37, *P* < 0.001, 95% CI [−0.48, −0.26]) and Black targets (*b* = −0.21, *P* = 0.002, 95% CI [−0.35, −0.08]). The two indices of mediated moderation were both negative and significant (Asian stereotypes: index of mediated moderation [IMM] = −0.06, 95% CI [−0.11, −0.01]; Black stereotypes: IMM = −0.03, 95% CI [−0.08, −0.01]), indicating that writing about the ongoing threat was associated with reduced intergroup bias more strongly under deliberative than intuitive processing. Specifically, such negative indirect effects were stronger under deliberative processing (Asian stereotypes: *b* = −0.11, 95% CI [−0.17, −0.06]; Black stereotypes: *b* = −0.06, 95% CI [−0.12, −0.02]) than under intuitive processing (Asian stereotypes: *b* = −0.05, 95% CI [−0.07, −0.03]; Black stereotypes: *b* = −0.03, 95% CI [−0.05, −0.01]).

Next, we tested whether these effects were further moderated by multicultural experience. A writing topic × depth of processing × multicultural experience interaction on integrative complexity was observed (*b* = 0.16, *P* < 0.001, 95% CI [0.10–0.22]). Writing about the ongoing threat (vs. nonthreat) increased integrative complexity most strongly under deliberative processing (*F*(1, 407) = 45.85, *b* = 0.33, *P* < 0.001). This interaction emerged only among participants high (+1 SD) in multicultural experience (*b* = 0.62, *P* < 0.001, 95% CI [0.51–0.74]) and not among participants low (−1 SD; *b* = 0.06, *P* = 0.26, 95% CI [−0.05, 0.17]).

**Figure 3:**
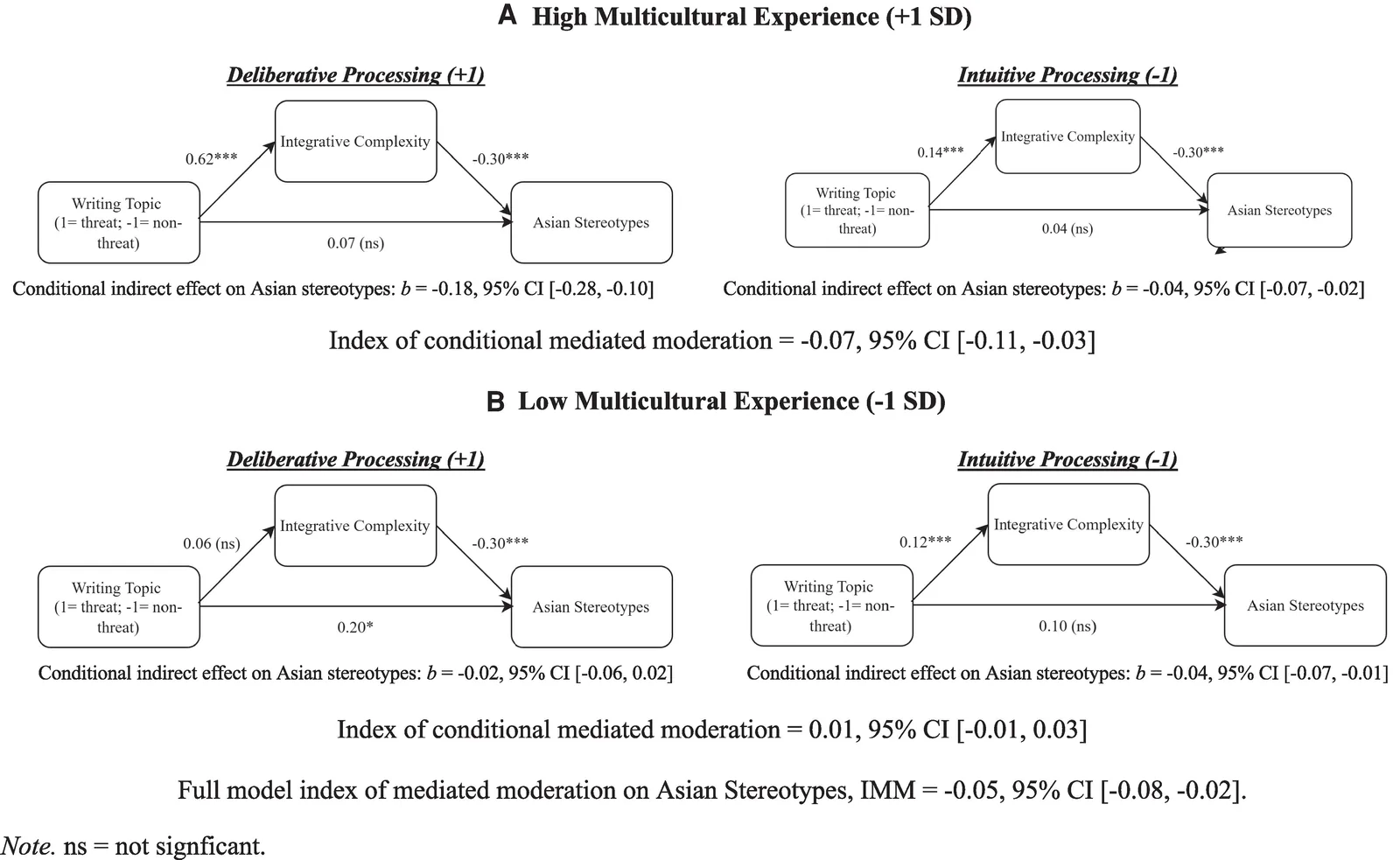
The conditional indirect effects of writing topic (threat vs. nonthreat) by depth of processing (deliberative vs. intuitive) on Asian stereotypes through integrative complexity, at (a) high (+1 SD) and (b) low (−1 SD) levels of multicultural experience, experiment 2.

**Figure 4:**
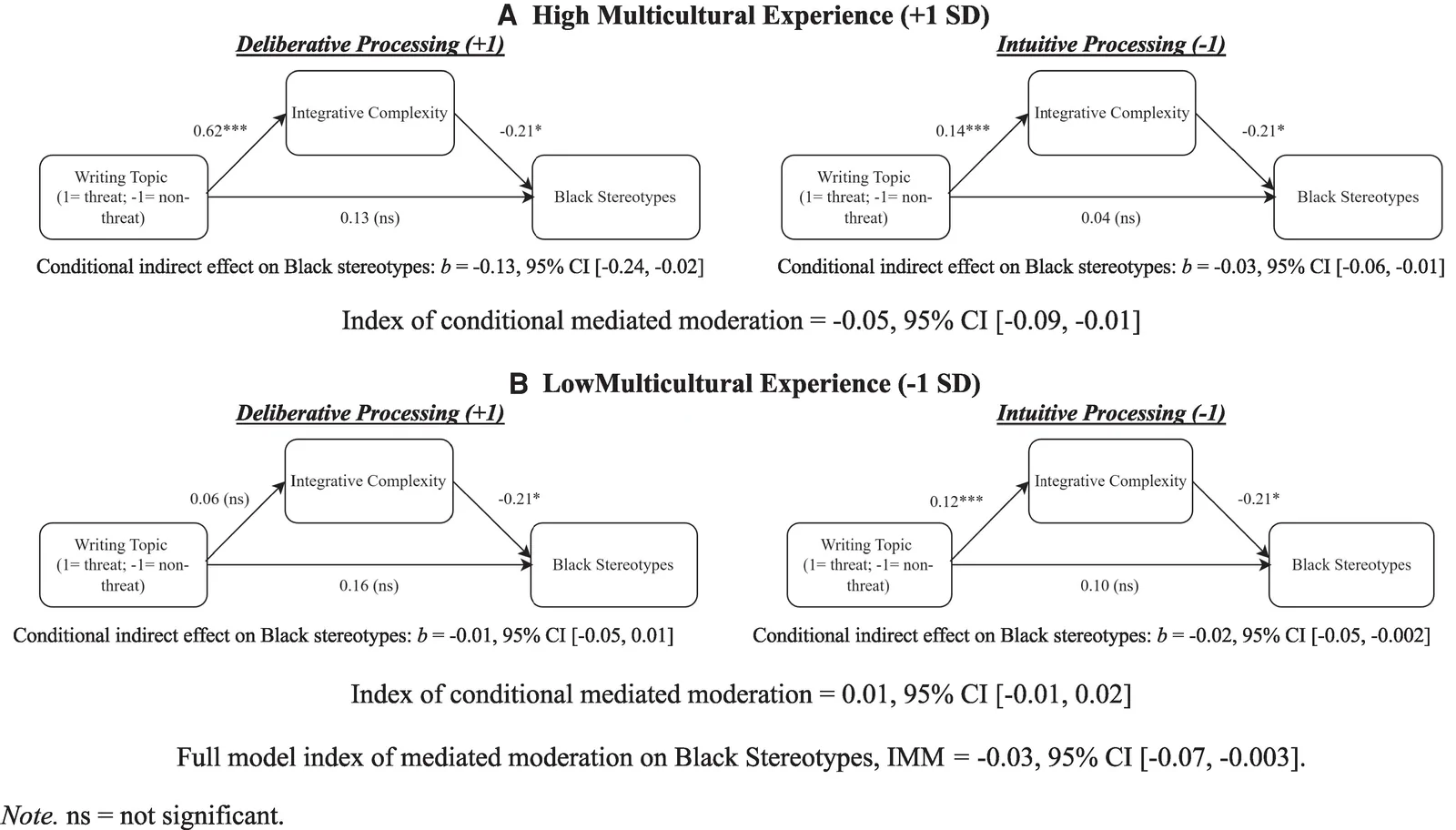
The conditional indirect effects of writing topic (threat vs. nonthreat) by depth of processing (deliberative vs. intuitive) on Black stereotypes through integrative complexity, at (a) high (+1 SD) and (b) low (−1 SD) levels of multicultural experience, experiment 2.

Analyses of intergroup bias yielded the same pattern. The indices of mediated moderation were significant for both Asian stereotypes (IMM = −0.05, 95% CI [−0.08, −0.02]) and Black stereotypes (IMM = −0.03, 95% CI [−0.07, −0.003]). As shown in Figs. 3 and 4, when multicultural experience was high (+1 SD), the indirect effect of writing on intergroup bias via integrative complexity was significantly strongest under deliberative processing (Asian stereotypes: *b* = −0.18, 95% CI [−0.28, −0.10]; Black stereotypes: *b* = −0.13, 95% CI [−0.24, −0.02]). No such effect emerged when multicultural experience was low (Asian stereotypes: IMM = 0.01, 95% CI [−0.01, 0.03]; Black stereotypes: IMM = 0.01, 95% CI [−0.01, 0.02]).

For descriptive completeness, we probed the writing topic × multicultural experience interaction on intergroup bias. The interaction was significant for Asian stereotypes (*b* = −0.09, *P* = 0.03, 95% CI [−0.17, −0.01]) but not for Black stereotypes (*b* = −0.05, *P* = 0.28, 95% CI [−0.15, 0.04]). Among participants low (−1 SD) in multicultural experience, writing about the threat (vs. nonthreat) predicted greater endorsement of Asian stereotypes (*b* = 0.12, *P* = 0.02, 95% CI [0.02–0.21]). No significant association emerged among participants high (+1 SD) in multicultural experience (*b* = −0.03, *P* = 0.52, 95% CI [−0.13, 0.07]).

## General discussion

The CTR model proposes that threat-induced intergroup bias can be regulated by targeting the cognitive architecture of social judgment through deliberative processing, conditional on cognitive preparedness. Across three experiments conducted during armed conflict, societal unrest, and a global pandemic, deliberative processing of an ongoing threat increased integrative complexity and reduced negative attitudes toward outgroups associated with the threat, primarily among individuals with greater multicultural experience, whereas these benefits did not emerge—and in some cases reversed—among individuals lower in multicultural experience. These findings show that the pathway from threat to tolerance is conditional: deliberation can engage complex reasoning under threat, but its effectiveness depends on individuals’ capacity to translate deliberation into integratively complex thought.

At the theoretical level, this work articulates, to our knowledge for the first time, a conditional architecture for reducing intergroup bias under ongoing societal threat. Rather than presenting a one-size-fits-all intervention, the CTR model addresses recent calls for personalization in intergroup interventions (56) by specifying when, and for whom, deliberative processing is effective in the face of threat. Such frameworks call for identifying the individual parameter on which effectiveness depends, the mechanism linking that parameter to outcomes, and the intervention itself. The CTR model supplies multicultural experience, integrative complexity, and deliberative processing of ongoing threat, respectively. Beyond its applied implications, the model also extends recent dual-process accounts of human sociality (57) into the intergroup domain. Bias toward a threat-associated outgroup can be conceived as a self-preservative response, whereby the ingroup is symbolically and physically protected by derogating the group perceived as the source of danger; the CTR model specifies the conditions under which deliberation can override this defensive response. In doing so, it integrates research streams that have largely developed in parallel—dual-process theories of social cognition, research on threat and intergroup bias, work on multicultural experience, the expressive-writing literature, and the integrative-complexity research—consistent with the call for theoretically integrative approaches to bias reduction (58).

The CTR model also speaks directly to the applied literature on intergroup bias reduction. Most established bias-reduction strategies, such as intergroup contact, require direct engagement with outgroups (13–15), yet active threat can make such engagement especially difficult precisely because defensive responses, avoidance, and bias toward threat-associated groups are heightened. The CTR model instead shifts attention from promoting intergroup contact to regulating the cognitive processes that shape social judgments under threat. Our findings show that restoring integrative complexity can reduce bias toward threat-associated groups even without direct intergroup contact, highlighting cognitive regulation as an alternative route to improving intergroup relations in precisely the contexts where hostility is likely to escalate and conventional contact-based approaches may be hardest to implement.

Our findings also extend research on mortality salience and terror management theory, which shows that when existential or societal threat remains active yet unprocessed, individuals tend to rely on simplified, defensive cognition that heightens intergroup bias (59, 60). Large-scale evidence from crises, such as the COVID-19 pandemic and the war in Ukraine, similarly links sustained threat to increased intolerance and attitude polarization (1, 61). The control conditions across our studies—those who were not prompted to reflect on the ongoing threat they had been experiencing—demonstrated this defensive pattern. Prior work suggests that consciously engaging with threatening experiences can help individuals regulate uncertainty and distress (62–64). We extend this logic by showing that such engagement attenuates intergroup bias, conditional on individuals’ capacity to translate deliberation into integratively complex thought.

The moderating role of multicultural experience identified here connects to a broader literature on cultural experience and cognition. Within the CTR model, multicultural experience functions not as a direct influence on bias but as the cognitive preparedness that allows deliberation to translate into integratively complex reasoning. This insight extends the literature demonstrating that multicultural experience fosters complex cognition across domains, facilitating creativity, leadership, and innovation (41, 42, 52, 65–67). Our findings suggest that multicultural experience does not only support complex thinking but also enhances responsiveness to interventions that engage higher-order cognition under threat. Practically, the CTR model suggests that deliberative processing of ongoing threat may be promising as a low-cost intervention in culturally diverse classrooms, workplaces, and peace-building contexts, particularly when these contexts make individuals’ prior multicultural experiences salient and thereby increase the accessibility of cognitive habits needed to translate deliberation into integratively complex thought. This possibility remains to be tested directly.

The present findings also advance research on expressive writing by clarifying its role as a cognitively targeted intervention under societal threat. Expressive writing has traditionally been studied for its intrapersonal benefits, including improvements in health, emotional regulation, and well-being (25, 26). The current work extends this literature by showing that expressive writing about ongoing threat can also shape intergroup judgments during societal threat by enabling higher-order cognitive processing, although these benefits are conditional on the topic engaged and on individuals’ cognitive preparedness.

Several specific features of the present design nevertheless bound what the studies can establish. The magnitude of the effects warrants careful interpretation. The predicted bias reduction effects consistently emerged in the conditional indirect pathway among individuals with the cognitive preparedness to translate deliberation into integratively complex thought in all experiments; however, the total and direct effects of threat-writing on bias varied across studies. Note that the CTR model does not anticipate uniform bias reduction; the conditional pattern is the model's signature prediction, and is consistent with a broader argument that intergroup interventions are unlikely to be uniformly effective across heterogeneous samples and that theoretically specified person-by-intervention fit is a more appropriate framework within which to evaluate intervention effectiveness (56, 58).

A further set of considerations concerns what our measures can establish about the proposed mechanism. Integrative complexity was coded from the same essays produced by the writing manipulation, so we cannot fully separate a general capacity for regulated cognition from features of reasoning specific to the assigned topics. We note, however, that integrative complexity indexes the structure of reasoning rather than the position taken. Essays written about the same threat topic varied substantially in complexity, with this variation depending on the multicultural experience of the writers and, in experiment 2, on how they were instructed to write. Effects driven by the content or style of the assigned topics would not be expected to vary in this way. Because our instructions directed participants to a designated topic, the present design was not constructed to examine variation in what participants chose to write about. Future study designs that permit such variation, paired with analyses of linguistic content, would help identify what features of writing carry the effect.

The path from integrative complexity to intergroup bias also warrants caution. Writing topic and depth of processing were randomly assigned, so their effects on integrative complexity are causally identified; however, the association between integrative complexity and bias is not. A purely dispositional account would not predict that randomly assigned writing topic shifts integrative complexity, that the magnitude of this shift depends on multicultural experience and depth of processing, or that lower bias is most consistently concentrated among outgroups associated with the focal threat rather than generalized across outgroups (see Supplementary material). Nor could preexisting differences in bias explain a shift in integrative complexity following random assignment. Integrative complexity is therefore responsive to experimental manipulation, and the conditional and outgroup-specific structure of the findings does not support a dispositional interpretation. That said, this does not eliminate the possibility that stable individual differences contribute to the observed association between integrative complexity and bias. Establishing the causal role of integrative complexity on bias reduction would require designs that manipulate it directly or assess it before and after the writing task.

A related consideration concerns the size of these effects. The modest effects observed in bias reduction are consistent with the broader prejudice-reduction literature, in which interventions typically produce only small shifts even under favorable conditions (58). What distinguishes the present case is that these effects emerged under ongoing societal threat: conditions in which the more typical trajectory is bias escalation rather than attenuation of intergroup bias (68–70). Detecting any bias reduction against this baseline of escalation is meaningful as any reduction in bias can represent an initial step toward enabling conversations that might address perceived intergroup tension. The practical reading of these effects is also bounded by the cost–benefit profile of the intervention itself. Compared with more resource-intensive treatments, brief, low-cost, scalable interventions can be practically meaningful even with small effect sizes, because their value depends on the ratio of effect to deployment cost rather than on absolute magnitude alone (26). A writing intervention that requires no specialized training can produce improvement on intergroup attitude under threat at scale fits this profile.

The conditional architecture of the CTR model predicts not only where the intervention works but where it may do harm. Among participants lower in multicultural experience, writing about the ongoing threat was associated with higher bias in two of the three experiments. The pattern was not uniform. It did not emerge in experiment 1b or for Black stereotypes in experiment 2, although its direction was consistent across the cases in which it appeared. This is also consistent with the mechanism the model specifies. Deliberative prompts invite elaboration, but for individuals lower in multicultural experience the cognitive habits that support integratively complex reasoning are less available. In such cases, deliberating about a threatening experience may foster rumination (37), and elaborating on it may amplify an existing biased interpretation rather than reduce it. Deliberation without the preparedness to use it is therefore not a neutral condition. As anticipated in frameworks for personalized intervention (56), our findings indicate that this intervention is not equally effective for all recipients, and that mismatch here is not merely ineffective. Practically, interventions of this kind should not be deployed indiscriminately. Whether these effects fade, persist, or compound over time is unknown, as is whether they can be averted by supporting deliberation more directly, for example by preceding it with an intervention that supplies the alternative perspectives that participants higher in multicultural experience appear to bring on their own.

That said, we view these findings as a first demonstration of the CTR architecture rather than as a complete account of its reach. Establishing that the conditional pathway operates across three real-world crisis contexts contributes to advancing a theoretical framework and a novel approach to understanding intergroup relations, and thus bias reduction. Yet, many questions remain for future investigation. First, the expressive-writing intervention was brief and administered only once. Although this single-session design allowed us to examine the immediate effects of deliberative processing on intergroup evaluations, it limits our ability to draw conclusions about the durability of these effects over time. While the broader expressive-writing literature documents durable intrapersonal effects from brief writing (26–28, 71, 72), whether expressive writing produces durable shifts in intergroup judgment is an open question that our single-session design cannot answer. Future research should examine whether repeated or sustained deliberative interventions (e.g. delivered across multiple sessions or embedded within ongoing intergroup encounters) can maintain or stabilize changes in cognitive processing and intergroup judgment.

Second, unlike much of the prior work on deliberative processing, cognitive capacity, and intergroup bias, which has relied on laboratory threat primes, the CTR model was tested with participants embedded in three sustained real-world crises. This ecological embedding answers calls for more field-based intergroup research (58), but it comes with trade-offs for experimental control. Although participants were randomly assigned across conditions, field studies in real-world settings cannot fully rule out extraneous influences. Relatedly, our outcomes were explicit self-reports of intergroup judgment rather than behavioral measures. We therefore cannot determine whether the observed shifts in judgment would translate into downstream behavior. Consequential decision tasks offer a tractable starting point. Prior work on multicultural experience showed that greater multicultural exposure reduced discrimination in a simulated hiring task (12). Analogous paradigms, including resource allocation and willingness to enter intergroup contact, could establish whether regulated cognition under threat shapes behavior as well as judgment, and administering them after a delay would address durability at the same time.

Third, although multicultural experience consistently moderated the effectiveness of deliberative processing, this individual difference was measured rather than manipulated. Our study was designed to investigate deliberative processing rather than to isolate the effects of multicultural experience. Thus, we did not directly test whether multicultural experience causally shapes responsiveness to deliberative interventions, although prior longitudinal and experimental work suggests that it can causally increase integrative complexity (12, 41). A related consideration is that multicultural experience is a composite index whose components might covary with other individual differences. Notably, prior work has shown its effects on integrative complexity to hold over and beyond intelligence, personality, and academic performance (39–43), and our own results were unchanged when demographic and dispositional covariates were included, including Big Five personality traits and political orientation (see Supplementary material). Such factors may nonetheless contribute to the pattern we observe. Future work manipulating multicultural experiences directly would help establish its causal role.

Finally, because our samples consisted of majority-group members, it is unclear whether the same mechanisms operate among minority populations, whose experiences of threat and intergroup dynamics may differ. Chronic exposure to threat, asymmetric power relations, and identity-relevant concerns (73) may shape both their baseline intergroup cognitions and responsiveness to deliberative prompts. Establishing whether and how the CTR architecture extends to these populations is an important direction for future work.

## Conclusion

Intergroup bias remains one of the most persistent challenges of contemporary societies, with prejudice and intensifying polarization posing serious risks to social cohesion. The CTR model specifies when threat-induced rigidity can be countered, even when societal threat remains acute. In an era of sustained societal threat, this model identifies not a cure-all, but a psychologically precise and conditional point of leverage, revealing when deliberative openings for tolerance can be fostered.

## Supplementary Material

pgag295_Supplementary_Data

## Data Availability

All raw data and analytic syntax supporting the findings of this study are publicly available via the Open Science Framework (OSF) repository at https://osf.io/6pb2s/overview?view_only=da3dbb48c3dc4fed9e536f9c088ba11a.
