# Supplementary material for: A conditional threat-regulation model: When deliberative processing of ongoing threat reduces intergroup bias
Source: PNAS Nexus. 2026 Sep 1;5(9):pgag295. doi: 10.1093/pnasnexus/pgag295 (PMC13575688; doi:10.1093/pnasnexus/pgag295)
Supplement: Supplementary file 1 [file pgag295_supplementary_data.pdf]

A Conditional Threat-Regulation Model: When Deliberative Processing of Ongoing Threat Reduces Intergroup Bias

Supplemental Materials

Table of Contents

Overview of Experiments and Supplemental Materials *2*
Study Timelines and Context *3*
Materials, Methods and Robustness Tests *4*
Experiments 1a and 1b 4
Experiment 2 12
Exploratory Coding: Descriptive Content Coding of Essays *16*
Coding scheme 16
Results 16
Model Diagnostics and Sensitivity Analyses *19*
Table S1. Correlations of key variables *24*
Table S1a. Correlations of key variables in Experiment 1a 24
Table S1b. Correlations of key variables in Experiment 1b 25
Table S1c. Correlations of key variables in Experiment 2 26
Table S2. Summary of key analyses *27*
Table S2a. Experiment 1a 27
Table S2b. Experiment 1b 28
Table S2c. Experiment 2 29
Table S2d. Sensitivity analyses results excluding influential observations in Experiment 1b (N = 117) 31
Table S2e. Sensitivity analyses results excluding influential observations in Experiment 2 (N = 402) 32
Exploratory Analyses *34*
Additional measures not reported in main text—Experiment 1a 34
Support for peace 34
Endorsement of Russian and Ethiopian stereotypes 35
Additional measures not reported in main text—Experiment 1b 36
Additional measures not reported in the main text—Experiment 2 36
Attitudes toward republicans and democrats 36
Table S3. Survey Questions and Item Order *38*
Table S3a. Survey Questions and Item Order, Experiment 1a 38
Table S3b. Survey Questions and Item Order, Experiment 1b 42
Table S3c. Survey Questions and Item Order, Experiment 2 45
References *47*

Overview of Experiments and Supplemental Materials

These supplemental materials provide comprehensive methodological, analytic, and robustness information for all experiments reported in the main text. They include detailed descriptions of study materials, procedures, measures, coding of integrative complexity, exclusion criteria, and additional analyses not reported in the main article. As reported in the main text, we test the Conditional Threat-Regulation (CTR) Model with two exploratory experiments (Experiments 1a and 1b), followed by a preregistered experiment (Experiment 2). Given the time-sensitive nature of the crises, sample sizes in Experiments 1a and 1b were necessarily constrained by the active duration of the events. We therefore used their observed effect sizes to plan Experiment 2. Across studies, we report all data exclusions (if any), manipulations, and measures, and we applied consistent exclusion criteria by retaining only majority-group participants in each context and excluding participants who failed instructions (e.g., leaving measures blank or writing gibberish) or produced unscorable essays. All studies were approved by our universities' institutional review board.

Tables S1a–S1c report correlations among key variables for Experiments 1a, 1b, and 2, respectively. Tables S2a–S2c present full bootstrapped moderated mediation results corresponding to the primary models tested in each experiment. Table S2d-S2e report the corresponding estimates from sensitivity analyses excluding observations identified as influential by residual-based and leverage-based criteria.

Additional exploratory analyses examining outcomes not central to the CTR Model are reported in subsequent sections. Tables S3a-3c present all survey questions for each experiment in the order they appeared to participants.

Study Timelines and Context

All three experiments were conducted while the focal societal threat was ongoing. Experiment 1a was conducted in Israel between 21 and 26 August 2014, during the final week of a 50-day armed conflict (8 July–26 August 2014), with data collection ending when an open-ended ceasefire took effect. Experiment 1b was conducted in Hong Kong SAR between 6 and 12 November 2014, during a 79-day social unrest (26 September–15 December 2014). Experiment 2 was conducted in the United States on 26 May 2020, during the first major U.S. wave of COVID-19. Data collection took place the day after the killing of George Floyd in Minneapolis. The national protest movement had not yet emerged at the time of data collection and escalated in the days that followed; no essay in the sample referenced George Floyd or the Black Lives Matter movement (0%).

Materials, Methods and Robustness Tests

(expanded details not reported in main text)

Experiments 1a and 1b

Participants

We aimed to recruit as many participants as possible during the armed conflict and the social unrest, given that the situations were time-sensitive. For Experiment 1a, given time and budget constraints, we aimed to recruit 90 participants. We stopped data collection once a cease-fire agreement was reached. Consequently, 82 participants from an Israeli university participated in the study in exchange for $12.5. Following the exclusion criteria reported in the overview section, seven Palestinian-Israeli participants were not included in the analyses. The final sample consisted of 75 Jewish-Israeli students (53.3% women; $M_{age} = 26.17$, $SD = 4.94$).

For Experiment 1b, 158 undergraduate participants from a Hong Kong university took part in the study for course credit. Based on the same criteria, 32 participants who were not Hong Kong Chinese were excluded from the analyses. One participant was excluded for not completing all measures and one was excluded due to unscorable writing, yielding a final sample of 124 Hong Kong Chinese participants (50.8% women; $M_{age} = 19.07$, $SD = .70$; one participant did not report age).

Procedure and session structure

Participants were informed at the outset that they would take part in two studies examining different psychological experiences and attitudes, described as conducted by different researchers but administered together for convenience. Both parts were completed in a single online session, with the second set of measures following immediately after the writing task. No measures were collected at a later date, and there were no follow-up assessments.

Materials

Writing Task. All participants were experiencing ongoing societal threat at the time the studies were conducted. They were randomly assigned to one of two writing conditions. In the *Threat Condition*, they wrote about their experiences with the ongoing threatening event (i.e., armed conflict in Experiment 1a and social unrest in Experiment 1b; coded as "1"). In the *Non-Threat Condition*, they wrote about their last dental visit (coded as "-1"), which is a common control topic in threat-related research (1). In both conditions, participants received the same deliberative processing instructions; only the writing topic varied.

All participants received the standard writing instructions that encouraged deliberative processing of the assigned topic (2). Specifically, in Experiment 1a, in the threat condition, participants read: "We would like to understand what you have been through in recent weeks during Operation Protective Edge. Try to experience the event again in your imagination and describe in as much detail as possible what you experienced, how you felt, and what you thought about. Try to write about 250 words and to devote at least 5 minutes to completing the task. Please note you cannot proceed in the survey until the 5 minutes have elapsed." In the non-threat condition they were given identical instructions, with the first sentence replaced by "We would like to understand what you have been through during your last visit to the dentist".

In Experiment 1b, in the threat condition, participants were asked to write about the social unrest and read: "Write down your experiences. Write down what you saw and how you felt (based on your personal experience, social media posting, or news media reports). Try to relive the experience again in your imagination. Describe the experience with as much detail as possible, taking into account the following questions: What did you see? How did you feel? What did you think about? Try to write at least 2–3 paragraphs and to devote at least 5 minutes to this task." In the non-threat condition, participants read: "Write an experience about your last

visit to a dentist," followed by the same relive-and-describe instructions and the same length and duration request.

Mean essay length was (M = 266.86, SD = 166.29) words in the threat condition and (M = 205.36, SD = 95.98) in the non-threat condition in Experiment 1a, and (M = 188.40, SD = 78.04) and (M = 141.43, SD = 58.34) in Experiment 1b. Controlling for word count, the pattern and significance of the results remained substantively unchanged in both experiments.

Task equivalence across conditions. To ensure that participants experienced the writing task similarly across conditions, after the task, we asked them to indicate (a) how hard the task was, (b) how much effort they put into the task, (c) how tiring the task was, and (d) how much they liked the task. Participants also rated both their positive (Experiment 1a: $\alpha = 0.75$; Experiment 1b: $\alpha = 0.93$) and negative emotions (Experiment 1a: $\alpha = 0.87$; Experiment 1b: $\alpha = 0.93$) after the writing.

Multicultural experience. In line with past research, we assessed multicultural experience with the Multicultural Experience Survey (MES) (4–7) which provides a summation score of a variety of experiences respondents have of interacting with people and/or elements of different cultures (3). In Experiment 1a, we used the full scale which measured length of time lived in Israel and other regions (Item 1), level of exposure to non-Israeli mainstream cultures on a 10-point scale (Item 2), number of foreign languages spoken (Item 3; participants could list up to four languages), parents' places of birth (Items 4 and 5); and the nationality of participants' five favorite cuisines (Item 6), friends (Item 7), and musicians (Item 8). Following Leung and Chiu (4), all items were given equal weight. For Experiment 1b, multicultural experience was measured and coded with the same scale except that the region was replaced with Hong Kong SAR instead of Israel in the relevant items. Items 4 and 5 about parental places of birth were accidentally omitted during programming in Experiment 1b.

As in previous research (4–7), we created the multicultural experience measure by rescaling the items so that each ranged from 0 to 1. Specifically, we estimated the amount of time lived *outside* Israel/Hong Kong SAR as a proportion of a person's age (Item 1), divided level of exposure by 10, the maximum scale response (Item 2), tallied the number of foreign languages spoken and divided by the maximum of four (Item 3), and coded whether participants' father and mother were born outside of Israel / Hong Kong SAR (Items 4 and 5) and divided the sum by 2. We further counted the number of listed restaurants that served non-local cuisines (Item 6), the number of foreign close friends (Item 7), and the number of foreign musicians (Item 8), and divided each by the maximum of five. The sum of the rescaled items forms our measure of multicultural experience.

Intergroup bias. We measured intergroup bias using established measures of negative attitudes toward the outgroup associated with the ongoing threatening event, with higher scores indicating greater intergroup bias. Specifically, in Experiment 1a, we created a composite of two established measures used in past research to assess negative attitudes toward Palestinian people. First, Halperin et al's (5) 3-item measure was used to assess general attitudes toward Palestinians with higher scores indicating more negative attitude ($\alpha = 0.87$). Second, negative stereotypes of Palestinian people were assessed by using Bar-Tal and Teichman's (6) 6-item measure with higher scores indicating more negative stereotypes toward Palestinian people ($\alpha = 0.91$). The scores of the two measures were highly correlated. Thus, they were averaged to create an overall composite ($\alpha = 0.92$). Separate analyses of each component yielded the same pattern of results as the composite score; hence, we report results only for the composite. To disguise the purpose of the study, these measures appeared as part of a larger questionnaire that examined social perceptions. The results of the measures that were not related directly to the study (e.g., negative

stereotypes towards Russians and Ethiopians) are presented in subsequent sections of the supplemental materials.

In Experiment 1b, during the social unrest in Hong Kong SAR, Mainland Chinese became discrimination targets (7, 8). We assessed negative attitudes towards Mainland Chinese using the same 20-item measure reported in Tadmor et al. (9) and adapted from Hong et al. (10, 11) with higher scores indicating higher intergroup biases. The measure captured prejudice and endorsement of stereotype toward Mainland Chinese, as well as the intention to avoid contact. All 20 items were averaged to create the composite ($\alpha = 0.91$). No additional measures were included in Experiment 1b.

Integrative complexity. The assessment of integrative complexity indexes the structure of thought with a 7-point scale where 1 reflects the absence of both differentiation and integration, 3 reflects the presence of differentiation but the absence of integration, 5 reflects the presence of both differentiation and integration, and 7 indicates differentiation as well as specification of higher order integrative principles. Even numbers (i.e., 2, 4, and 6) are assigned as transitional scores when a response implies the next higher level of complexity but does not explicitly meet the scoring criteria for that level. Notably, some texts are deemed as unscorable because "the author's rule structure for drawing inferences or making decisions is not evident" (p.10; (12). These unscorable texts need to be excluded from analysis. Crucially, integrative complexity is scored on the basis of how the author structures their reasoning, not what they argue for: the same position can be expressed at any level of complexity, and opposing positions can be expressed at the same level (14).

Two pairs of trained coders, who were blind to the purpose of the studies, independently assessed the integrative complexity of the essays, with high inter-judge reliability (Experiment

1a: α = 0.92; Experiment 1b: α = 0.91). No essays were considered unscorable by the coders in Experiment 1a, but one was considered so in Experiment 1b.

Statistical Analysis

All path coefficients reported throughout the manuscript are unstandardized. The 95% confidence intervals for direct effects were derived from the *t*-distribution, based on the standard error of each regression coefficient. The 95% confidence intervals for indirect and conditional indirect effects were percentile bootstrap intervals based on 5,000 resamples, computed using Hayes's (13) PROCESS macro.

To improve interpretability, and get meaningful main effects, following Hayes' (13) suggestions, multicultural experience was mean-centered and the writing topic variable was contrast coded such that a score of "1" was assigned for writing about ongoing threat and a score of "-1" was assigned for writing about the non-threat topic. The CTR Model focuses on the potential of deliberative processing of ongoing threat to increase integrative complexity during ongoing threat, with decreasing intergroup biases as a downstream implication. Thus, consistent with the logic of the CTR model, we focused on the direct effects of writing topic manipulation on integrative complexity and their indirect effects on intergroup biases. Moreover, given our theoretical focus on the effects of the writing topic manipulation on integrative complexity, we only included participants who provided complete data in all analyses (i.e., had a scorable essay). Crucially, when we conducted analyses for the full samples (including those who did not have a scorable essay), we found similar patterns of results as those reported in the text.

Task Equivalence and Robustness Tests

We checked task equivalence for the two writing conditions in Experiments 1a and 1b. As expected, independent sample t-tests revealed no systematic differences between conditions in terms of task difficulty (Experiment 1a: $t(73) = 1.29$, $p = 0.20$, $d = 0.30$; Experiment 1b: $t(122)$

= 0.26, *p* = 0.80; *d* = 0.05), task effort (Experiment 1a: *t*(73) = 1.21, *p* = 0.23, *d* = 0.28; Experiment1b: *t*(122) = 0.79, *p* = 0.43; *d* = 0.14), and post-task positive emotions (Experiment 1a: *t*(73) = 0.95, *p* = 0.35, *d* = 0.22; Experiment 1b: *t*(122) = 0.81, *p* = 0.42; *d* = 0.15). Notably, whereas no differences were found for post-task negative emotions and for how tiring the task was among Experiment 1b participants (post-task negative emotions: *t*(122) = 0.98, *p* = 0.33, *d* = 0.18; task tiring: *t*(122) = -1.53, *p* = 0.13, *d* = -0.28), Experiment 1a participants reported marginally more post-task negative emotions (*t*(73) = 1.88, *p* = 0.07, *d* = 0.43) and being significantly more tired when writing about the ongoing threatening event (*t*(73) = 2.04, *p* = 0.05; *d* = 0.47). Specifically, participants felt more tired (*M* = 2.42, *SD* = 1.18) and more negative emotions (*M* = 2.06, *SD* = 0.90) after writing about the ongoing armed conflict (*n* = 36) than about a dental visit (*n* = 39; tiredness: *M* = 1.90, *SD* = 1.02; negative emotions: *M* = 1.71, *SD* = 0.76). These results are consistent with past research showing that an increase in negative emotions following expressive writing exercises is common (14). Finally, whereas Experiment 1a participants did not differ in how much they liked the writing task (*t*(73) = 1.56, *p* = 0.12; *d* = 0.36), Experiment 1b participants (*t*(122) = 5.06, *p* < 0.001, *d* = 0.91), liked the task more when writing about the ongoing societal unrest (*n* = 57, *M* = 3.00, *SD* = 1.10) than when writing about a dentist visit (*n* = 67, *M* = 2.03, *SD* = 1.03). Importantly, the pattern of the primary results remained unchanged when controlling for these differences.

We also conducted additional analyses to assess the robustness of our findings by including a set of theoretically relevant covariates. Specifically, we controlled for demographic and dispositional variables commonly included in prior research (15, 16), as well as established correlates of social tolerance (17, 18). These covariates comprised age, gender, Big Five personality traits (19), and conservative political orientation (20); in Experiment 1a, we

additionally controlled for the importance of religion (21). The pattern and significance of the results remained substantively unchanged with and without these controls.

Experiment 2

Participants

We based our sample size estimation on Cohen's f^2 effect sizes of the interaction term from Experiments 1a and 1b, which ranged from 0.07 to 0.08. Using G*Power for a linear multiple regression (fixed model, R^2 increase) and aiming for 95% power ($1 - \beta$) with $\alpha = 0.05$, the analysis indicated that a minimum sample of 186 participants would be needed. Because we were not aware of techniques at the time that could provide a sample size estimate for the full moderated mediation model, we targeted 500 White American participants living in the United States through the online data collection platform Prolific, providing a conservative margin for detecting the higher-order effects and buffering against anticipated exclusions. Participants were paid GBP 2.50 for completing the survey.

Out of the 500 participants recruited, and applying the same exclusion criteria used in Experiments 1a and 1b, 49 ethnic minority participants were excluded based on the majority-group criterion, 22 participants were excluded for not following instructions (e.g., writing gibberish) and 14 participants were excluded for unscorable writing tasks. The sample for the full analyses consisted of 415 participants (52.5% men; $M_{age} = 34.29$, $SD = 12.25$).

Procedure and session structure

The experiment was administered as a single session. The writing task and all subsequent measures were completed in that session, and there were no follow-up assessments.

Materials

Writing topic. As in Experiments 1a and 1b, all participants were experiencing ongoing societal threat at the time the studies were conducted. They were randomly assigned to write about either the threat (i.e., coronavirus pandemic; coded as "1") or a non-threat topic (i.e., last movie/TV program they had watched; coded as "-1"). In the deliberative threat condition,

participants read: "Write down your experiences during the ongoing COVID-19 outbreak. Try to relive the experience in your imagination. Describe the experience with as much detail as possible, taking into account the following questions: What did you see? How did you feel? What did you think about? Think deeply about how it affects you. Elaborate as much as possible. Try to write at least 2–3 paragraphs and to devote at least 3 minutes to this task." In the deliberative non-threat condition, the instructions were identical in structure, but referred to "the last movie/TV program you watched" and to reliving its scenes.

Depth of processing. Participants were randomly assigned to the *deliberative processing* or the *intuitive processing* condition. In the deliberative processing condition (coded as “1”), as in previous experiments, and as elaborated on above, participants were asked to deeply reflect on their thoughts and feelings about the assigned topic. In contrast, in the *intuitive processing* condition (coded as “-1”), we adopted a procedure that has been shown to “knock out” deliberative processing in the past (22–24). Participants were instructed to write down their first response to the writing topic as quickly as possible while completing a concurrent cognitive load task. Specifically, they performed the dot memorization task (25). The exact instructions in the intuitive processing condition were: “Before recalling and writing about an event, you will be presented with an image in the following page. The image shows a 9-box grid like the one shown on this page. Some of the boxes will be marked with 'X's. Your task is to remember the pattern of 'x' displayed in the grid as you will be asked to recall the pattern later on. The image will only be displayed for 2 seconds. Again, make sure you remember which boxes the 'X's are in." The grid was then displayed for two seconds under the heading "Memorize this…", after which participants read: "Remember to keep the 9-box grid pattern in mind. Next, write down your experiences during the ongoing COVID-19 outbreak (based on your personal experience, social media posting, or news media reports). Put down the first thing that comes to your mind."

Participants in the non-threat condition received the same text, referring instead to "the last movie/TV program you watched." After writing, participants completed a four-alternative recall check for the grid pattern. Participants in the intuitive conditions received no duration or length request, consistent with the instruction to record their first response. Past research shows that this dual-task procedure suppresses deliberative processing, ensuring participants' responses to the writing task reflect intuitive processing (22, 23, 26, 27).

Mean essay length was (M = 161.05, SD = 100.06) words in the deliberative conditions and (M = 43.46, SD = 48.51) words in the intuitive conditions, reflecting the difference in instructions described above. Controlling for word count, the pattern and significance of the results remained substantively unchanged.

Multicultural experience. We used the full eight-item Multicultural Experience Survey (4, 9, 28, 29).

Intergroup bias. The measures of intergroup bias assessed prejudice towards Asian and Black people because these two groups experienced a significant increase in discrimination during the outbreak of the coronavirus pandemic (30, 31). Specifically, Asian Americans became associated with the threat through media accounts of disease origin: COVID-19 was traced to China and framed in public discourse as a "Chinese virus", with blame generalizing beyond Chinese Americans to Asian Americans broadly through the perpetual foreigner stereotype and outgroup homogeneity effects (32, 33). Black Americans became associated with the threat through widely publicized evidence of substantially higher rates of COVID-19 mortality and morbidity (34), which had received extensive media coverage (35) by the time of our May 2020 data collection. Researchers documented greater intergroup competition, desired social distance, and perceived disease-transmission risk toward Black targets during this period of the pandemic (36–38). To measure intergroup bias against these two groups, following Tadmor et al. (9),

participants rated their agreement with six Asian stereotypes (α = 0.79) (39), and six Black stereotypes (α = 0.91) (40). Higher scores reflect stronger endorsement of stereotypes of these outgroups, and thus, higher intergroup bias. To disguise the focus of the study, we also measured attitudes toward political groups; these measures and results are reported in subsequent sections of the supplemental materials, and were not central to tests of the CTR Model.

Integrative complexity. Two trained coders, who were blind to the purpose of the study, independently assessed the integrative complexity of the writings and produced acceptable inter-judge agreement (α = 0.83). Fourteen essays were judged to be unscorable.

Statistical Analysis

Because Experiment 2 was pre-registered (https://aspredicted.org/blind.php?x=sh9zw8), we note an important clarification regarding analytic structure. In the preregistration, we specified multicultural experience as the predictor, the writing topic × depth of processing interaction as the moderator, and integrative complexity as the mediator. In the final analyses, we modeled writing topic and depth of processing as the independent variables, integrative complexity as the mediator, and multicultural experience as the moderator. Because the interaction term is mathematically identical under either specification, this refinement does not alter the statistical substance of our predictions. Instead, it more accurately reflects our theoretical logic: writing topic and depth of processing are the manipulated factors, integrative complexity is the proposed mechanism, and multicultural experience serves as the boundary condition. Accordingly, the analytic structure was refined for conceptual clarity while maintaining consistency with the preregistered hypotheses.

Exploratory Coding: Descriptive Content Coding of Essays

Integrative complexity coding scores the structure of reasoning, not the content of what is written. However, to explore what participants actually wrote about in the writing task and make the essay content transparent, we coded essays from all three experiments for three descriptive content features. They were coded as follows.

Coding scheme

Code 1: On-topic engagement. A binary code (0 = off-topic or non-responsive; 1 = on-topic, addressing the assigned topic). The code was applied to all essays in both threat and non-threat conditions, using the assigned topic as the referent. For Experiment 2, essays from the intuitive-processing conditions were coded using the same rubric; brief responses that are on-topic are expected by design and are not treated as failures of the manipulation.

Code 2: Outgroup mention. A two-step code. Step 1 classified whether the essay explicitly referenced the groups associated with the threat (0 = not mentioned; 1 = mentioned). Step 2, applied only when outgroup was mentioned. It classified the nature of the reference: G = geographic (e.g., Gaza); P = political entity (e.g., Hamas); or S = social group (e.g., Palestinians). When multiple references appeared in a single essay, the highest-intensity type was assigned (e.g., S > P > G).

Code 3: Multicultural content. A binary code (0 = absent; 1 = present) for whether the essay contained substantive references to the writer's own multicultural experience, including foreign-born or immigrant background, time lived or studied abroad, substantive foreign friends or family, multilingual navigation of cultural frames, or bicultural identity references.

Results

Experiment 1a: Armed conflict. In the threat condition, 100% of essays were on topic. Explicit mention of a threat-associated outgroup was present in 52.78% of threat-condition

essays; among these, 11.11% referenced a political entity (e.g., Hamas), 25% a geographic context (e.g., Gaza as location), and 16.67% a social category. In the non-threat condition, 100% of essays were on topic, but none mentioned a threat-associated outgroup. Across both conditions, explicit references to the writer's own multicultural experience appeared in 0% of essays.

Experiment 1b: Social unrest. In the threat condition, 100% of essays were on topic. Explicit mention of a threat-associated outgroup was present in 24.56% of threat-condition essays, and all referenced a political entity. Explicit references to the writer's own multicultural experience appeared in 1.75% of essays. In the non-threat condition, 100% of essays were on topic, but none mentioned a threat-associated outgroup, and none referenced the writer's own multicultural experience.

Experiment 2: Pandemic. In the threat condition, 99.5% of essays were on topic (the remaining 0.5% wrote about an off-topic subject and were retained in the analyses; the pattern of results was unchanged when these participants were excluded). Asian references appeared in 7.5% of threat-condition essays. Of these, 5% geographic, 1% political entity, and 1.5% social group; Black references appeared in 0% of essays as social group. Explicit references to the writer's own multicultural experience appeared in 2.5% of essays. In the non-threat condition, 99.07% of essays were on topic (the pattern of results was unchanged when the remaining 0.93% of participants were excluded). None mentioned the Asian outgroup, 0.93% mentioned the Black outgroup (all as social category references), and none referenced the writer's own multicultural experience.

Data was collected on May 26, 2020, incidentally the day after the murder of George Floyd. Thus, we also coded whether participants referenced Black Lives Matter or George Floyd; none did (0%).

Across all three experiments, participants mostly wrote as instructed. Importantly, although virtually all participants in the threat condition engaged with the focal threat, integrative complexity varied substantially across their essays. This pattern supports the distinction between the *content* of what participants wrote (i.e., the writing focus) and the *structure* with which they reasoned about that content (i.e., integrative complexity)—a distinction that is central to the theoretical logic of the CTR Model.

Model Diagnostics and Sensitivity Analyses

We report additional diagnostic and sensitivity analyses to assess model adequacy and address the role of sample size in the focal tests. Below, we report model diagnostics for the component regression models underlying the indirect and conditional indirect effects. Specifically, we examined residual distributions (41), variance inflation factors and tolerance values (42), Cook's distances (43), and leverage values (44, 45). These diagnostics did not indicate serious departures from normality, problematic multicollinearity, or evidence that the focal effects were driven by highly influential observations in ways likely to materially affect inference.

Residual distribution and normality. We inspected histograms and normal probability plots of standardized residuals for each component regression model. The residual distributions did not indicate serious departures from normality. For Experiment 1a, the largest standardized residual fell within the acceptable range (conventional threshold < 3) (46), suggesting that non-normality of residuals was unlikely to materially affect our focal conclusions. For Experiments 1b and 2, the largest standardized residual was 3.61 and 3.65, respectively, and three observations in Experiment 1b and four observations in Experiment 2 showed an absolute standardized residual greater than 3.29.

Multicollinearity. We examined variance inflation factors (VIFs) and tolerance values for all predictors, including interaction terms. For Experiment 1a, VIFs ranged from 1.00 to 1.36 and tolerance values from 0.74 to 1.00. For Experiment 1b, VIFs ranged from 1.00 to 1.47 and tolerance values from 0.75 to 1.00. For Experiment 2, VIFs ranged from 1.02 to 1.37 and tolerance values from 0.77 to 0.96. Across the component regression models, these values were acceptable by conventional thresholds (VIF < 10, tolerance > 0.20) (42). Collinearity was therefore not a serious concern in our moderated mediation models.

Influential observations. We examined Cook's distances and leverage values to assess whether the focal effects were driven by highly influential cases. The maximum Cook's distance was 0.10 in Experiment 1a, 0.11 in Experiment 1b, and 0.10 in Experiment 2; no case exceeded the conventional threshold of 1 (45). The maximum leverage value was 0.24 in Experiment 1a, 0.18 in Experiment 1b, and 0.12 in Experiment 2. The recommended cutoff for leverage values is $3(k+1)/n$ in small samples, where k is the number of predictors and n is the sample size (yielding cutoffs of 0.20 in Experiment 1a, 0.12 in Experiment 1b, and 0.07 in Experiment 2) (44). The results on leverage values identified zero influential observations in Experiment 1a sample, five influential observations in Expeirment 1b sample, and nine influential observations in Experiment 2 sample. Combining with the influential observations identified in residual distribution and normality tests, we therefore identified seven influential observations in Expeirment 1b and thirteen influential observations in Experiment 2. Leverage diagnostics identified no potentially influential observations in Experiment 1a, five in Experiment 1b, and nine in Experiment 2. After combining these results with those from the residual and influence diagnostics, we identified seven observations in Experiment 1b and thirteen observations in Experiment 2 for further examination. We then re-ran the regression analyses excluding these influential observations. As reported in Table S2d and Table S2e, the substantive conclusions remained unchanged: the focal interaction terms, conditional indirect effects, and indices of moderated mediation were consistent in direction and inference. These results suggest that the moderated mediation findings were unlikely to be driven by highly influential cases. The primary analyses therefore retained the full sample.

Sample-size context. As detailed in the *Statistical Analysis* section above, sample sizes for Experiments 1a and 1b were not determined by a prospective power analysis (data were collected as feasible during an ongoing threat context), whereas Experiment 2 was prospectively powered

based on the focal interaction observed in Experiments 1a and 1b. The prospective power analysis indicated a minimum required sample of 186 participants. Given the variations in the experimental context and the addition of the Depth of Processing factor in Experiment 2, we aimed to recruit more participants than the minimum required sample. The final sample comprised of 415 participants.

Sensitivity power analysis. To contextualize the analyses given the non-prospectively-determined sample sizes in Experiments 1a and 1b, we conducted sensitivity power analyses for the focal tests of the CTR Model. The model does not predict main effects on bias; it predicts a conditional indirect effect—bias reduction through integrative complexity, conditional on multicultural experience. Power for the focal moderated-mediation tests therefore depends jointly on (a) the interactions predicting integrative complexity (the writing topic × multicultural experience interaction in Experiments 1a and 1b, and the writing topic × depth of processing × multicultural experience three-way interaction in Experiment 2), and (b) the integrative complexity slope predicting intergroup bias, controlling for writing condition. This yields seven focal tests across the three studies: three interactions and four integrative complexity slopes. We computed the smallest effect size detectable at 80% power and $\alpha = 0.05$ (two-tailed) for each focal coefficient using G*Power's "Linear Multiple Regression (fixed model, R^2 increase)" routine, matching the routine used for the prospective power analysis in Experiment 2.

For the focal interactions predicting integrative complexity—the path that establishes the conditional structure—the smallest detectable partial f^2 given the obtained sample sizes at 80% power with $\alpha = 0.05$ was 0.108 in Experiment 1a, 0.064 in Experiment 1b, and 0.019 in Experiment 2. The observed coefficients yielded partial f^2 of 0.075 in Experiment 1a, 0.071 in Experiment 1b, and 0.065 in Experiment 2.

For the integrative complexity slopes predicting bias—the path through which the conditional reduction in bias operates—the smallest detectable partial f^2 given the obtained sample sizes at 80% power with $\alpha = 0.05$ was 0.108 in Experiment 1a, 0.064 in Experiment 1b, and 0.019 in Experiment 2 (across both target groups). The observed coefficients yielded partial f^2 of 0.228 in Experiment 1a, 0.092 in Experiment 1b, and 0.049 for Asian stereotypes and 0.016 for Black stereotypes in Experiment 2.

Across these seven focal tests, observed effects exceeded the 80%-power detection floor in five cases. The two that fell modestly below their floors but reached significance in inferential testing were: (i) the writing-topic × multicultural experience interaction in Experiment 1a (the smallest, exploratory study, conducted under field-collection constraints during ongoing armed conflict), and (ii) the integrative complexity slope predicting Black stereotypes in Experiment 2 (a small but significant effect, $t = -2.52$, consistent in direction with the model's prediction).

These results correspond to the conditional architecture the CTR Model predicts. These sensitivity analyses are intended to contextualize the detectable size of the focal component-regression effects rather than to serve as post hoc power tests of the indirect effects themselves, and we interpret the resulting conditional indirect effects with caution.

Specification sensitivity. We also conducted sensitivity analyses to examine whether the conclusions depended on analytic specification. The models were estimated both with and without covariates (covariates are described in the *Statistical Analysis* section above), and the substantive conclusions were consistent across specifications. In addition, we compared models using 5,000 versus 10,000 bootstrap resamples; the indirect effects, conditional indirect effects, and indices of moderated mediation showed the same substantive pattern across both bootstrapping resamples.

Together, these diagnostics and sensitivity analyses did not indicate serious model adequacy concerns. The focal mediation and moderated mediation conclusions were robust to checks of residual normality, multicollinearity, influential observations, covariate adjustment, and bootstrap resample size.

Table S1. Correlations of key variables

Table S1a. Correlations of key variables in Experiment 1a

	Writing Topic	Multicultural Experience	Integrative Complexity	Negative Attitudes toward Palestinians
Writing topic (1=Threat; -1=Non-Threat)	--			
Multicultural Experience	-0.01	--		
Integrative Complexity	0.28*	0.26*	--	
Negative Attitudes toward Palestinians	0.09	0.04	-0.40**	--

**. Correlation is significant at the 0.05 level (2-tailed).*
***. Correlation is significant at the 0.01 level (2-tailed).*

Table S1b. Correlations of key variables in Experiment 1b

	Writing Topic	Multicultural Experience	Integrative Complexity	Negative Attitudes toward Mainland Chinese
Writing topic (1=Threat; -1=Non-Threat)	--			
Multicultural Experience	0.01	--		
Integrative Complexity	0.25^{**}	0.37^{**}	--	
Negative Attitudes toward Mainland Chinese	-0.01	-0.27^{**}	-0.36^{**}	--

**. Correlation is significant at the 0.05 level (2-tailed).*

***. Correlation is significant at the 0.01 level (2-tailed).*

Table S1c. Correlations of key variables in Experiment 2

	Depth of Processing	Writing Topic	Multicultural Experience	Integrative Complexity	Stereotypes: Asians	Stereotypes: Blacks
Depth of Processing (1=Deliberative; -1=Intuitive)	--					
Writing topic (1=Threat; -1=Non-Threat)	-0.01	--				
Multicultural Experience	-0.06	-0.04	--			
Integrative Complexity	0.35**	0.30**	0.23**	--		
Stereotypes: Asians	0.02	0.07	-0.14**	-0.24**	--	
Stereotypes: Blacks	-0.05	0.08	-0.05	-0.12**	0.57**	--

**. Correlation is significant at the 0.05 level (2-tailed).*

***. Correlation is significant at the 0.01 level (2-tailed).*

Table S2. Summary of key analyses

Table S2a. Experiment 1a

Hayes Process Model 8	*b*	*SE*	*t*	*p*	95% CI LL	95% CI UL
**Writing Topic (Threat vs. Non-Threat) × Multicultural Experience → Integrative Complexity**						
Writing Topic (1=Threat; -1=Non-Threat)	0.28	0.11	2.64	0.01	0.07	0.50
Multicultural Experience	0.44	0.14	3.14	0.002	0.16	0.73
Multicultural Experience × Writing Topic	0.33	0.14	2.30	0.02	0.04	0.61
**Writing Topic (Threat vs. Non-Threat) × Multicultural Experience → Integrative Complexity → Negative Attitudes toward Palestinians**						
Writing Topic (1=Threat; -1=Non-Threat)	0.26	0.13	2.01	0.05	0.002	0.52
Integrative Complexity	-0.55	0.14	-3.99	< 0.001	-0.82	-0.27
Multicultural Experience	0.16	0.17	0.92	0.36	-0.19	0.51
Multicultural Experience × Writing Topic	-0.23	0.17	-1.36	0.18	-0.57	0.11
**Indirect effect**						
High Multicultural Experience (+1 SD)	-0.30	0.11			-0.53	-0.08
Low Multicultural Experience (-1 SD)	-0.01	0.08			-0.19	0.15
Index of Mediated Moderation	-0.18	0.09			-0.35	-0.002

Table S2b. Experiment 1b

**Hayes Process Model 8**	***b***	***SE***	***t***	***p***	**95% CI**	
					**LL**	**UL**
**Writing Topic (Threat vs. Non-Threat) × Multicultural Experience → Integrative Complexity**						
Writing Topic (1=Threat; -1=Non-Threat)	0.24	0.08	3.07	0.003	0.09	0.40
Multicultural Experience	0.68	0.13	5.32	< 0.001	0.43	0.93
Multicultural Experience × Writing Topic	0.37	0.13	2.92	0.004	0.12	0.63
**Writing Topic (Threat vs. Non-Threat) × Multicultural Experience → Integrative Complexity → Negative Attitudes toward Mainland Chinese**						
Writing Topic (1=Threat; -1=Non-Threat)	0.07	0.06	1.10	0.27	-0.05	0.19
Integrative Complexity	-0.22	0.07	-3.30	0.001	-0.36	-0.09
Multicultural Experience	-0.18	0.11	-1.74	0.08	-0.39	0.03
Multicultural Experience × Writing Topic	-0.05	0.10	-0.52	0.60	-0.24	0.14
**Indirect effects**						
High Multicultural Experience (+1 SD)	-0.11	0.04			-0.20	-0.03
Low Multicultural Experience (-1 SD)	0.004	0.03			-0.06	0.05
Index of Mediated Moderation	-0.08	0.04			-0.16	-0.02

Table S2c. Experiment 2

**Hayes Process Model 12**	***b***	***SE***	***t***	***p***	**95% CI**	
					**LL**	**UL**
**Writing Topic (Threat vs. Non-Threat) × Depth of Processing (Deliberative vs. Intuitive) × Multicultural Experience → Integrative Complexity**						
Writing Topic (1=Threat; -1=Non-Threat)	0.24	0.03	9.31	< 0.001	0.19	0.29
Depth of Processing (1=Deliberative; -1=Intuitive)	0.27	0.03	10.53	< 0.001	0.22	0.32
Writing Topic × Depth of Processing	0.11	0.03	4.15	< 0.001	0.06	0.16
Multicultural Experience	0.27	0.03	8.61	< 0.001	0.21	0.33
Writing Topic × Multicultural Experience	0.17	0.03	5.59	< 0.001	0.11	0.23
Depth of Processing × Multicultural Experience	0.20	0.03	6.61	< 0.001	0.14	0.27
Writing Topic × Depth of Processing × Multicultural Experience	0.16	0.03	5.14	< 0.001	0.10	0.22
**Conditional Effects**						
Deliberative Processing: $b = 0.33$, $F(1, 407) = 45.85$, $p < 0.001$						
High Multicultural Experience (+1 SD)	0.62	0.06	10.77	< 0.001	0.51	0.74
Low Multicultural Experience (-1 SD)	0.06	0.05	1.13	0.26	-0.05	0.17
Intuitive Processing: $b = 0.01$, $F(1, 407) = 0.13$, $p = 0.71$						
High Multicultural Experience (+1 SD)	0.14	0.05	3.13	0.002	0.05	0.23
Low Multicultural Experience (-1 SD)	0.12	0.05	2.49	0.01	0.03	0.21

Table S2c. Experiment 2 (cont.)

Models	*b*	SE_{boot}	Bootstrap 95% CI	
			LL	UL
**Multicultural Experience × Writing Topic (Threat vs. Non-Threat) × Depth of Processing (Deliberative vs. Intuitive) → Integrative Complexity → Stereotypes**				
Model 12				
DV: Asian Stereotypes				
**Indirect Effects**				
**Index of mediated moderation**	-0.05	0.01	−0.08	−0.02
High Multiculturalism (+1 SD)				
Deliberative Processing	-0.18	0.05	−0.28	-0.10
Intuitive Processing	-0.04	0.01	−0.07	-0.02
Index of conditional mediated moderation	-0.07	0.02	−0.11	-0.03
Low Multiculturalism (-1 SD)				
Deliberative Processing	-0.02	0.02	−0.06	0.02
Intuitive Processing	-0.04	0.01	−0.07	-0.01
Index of Conditional Mediated Moderation	0.01	0.01	−0.01	0.03
DV: Black Stereotypes				
**Indirect Effects**				
**Index of mediated moderation**	-0.03	0.02	−0.07	−0.003
High Multiculturalism (+1 SD)				
Deliberative Processing	-0.13	0.06	−0.24	-0.02
Intuitive Processing	-0.03	0.01	−0.06	-0.01
Index of Conditional Mediated Moderation	-0.05	0.02	−0.09	-0.01
Low Multiculturalism (-1 SD)				
Deliberative Processing	-0.01	0.02	−0.05	0.01
Intuitive Processing	-0.02	0.01	−0.05	-0.002
Index of Conditional Mediated Moderation	0.01	0.01	−0.01	0.02

Note. Path coefficients are unstandardized estimates. *SE*s and *CI*s of indirect effects and IMMs were estimated with bootstrapping.

Table S2d. Sensitivity analyses results excluding influential observations in Experiment 1b (N = 117)

**Hayes Process Model 8**	***b***	***SE***	***t***	***p***	**95% CI**	
					**LL**	**UL**
**Writing Topic (Threat vs. Non-Threat) × Multicultural Experience → Integrative Complexity**						
Writing Topic (1=Threat; -1=Non-Threat)	0.20	0.07	2.78	0.01	0.06	0.34
Multicultural Experience	0.97	0.14	7.03	< .001	0.69	1.24
Multicultural Experience × Writing Topic	0.44	0.14	3.23	0.002	0.17	0.72
**Writing Topic (Threat vs. Non-Threat) × Multicultural Experience → Integrative Complexity → Negative Attitudes toward Mainland Chinese**						
Writing Topic (1=Threat; -1=Non-Threat)	0.05	0.06	0.89	0.37	-0.06	0.17
Integrative Complexity	-0.21	0.07	-2.86	0.01	-0.36	-0.07
Multicultural Experience	-0.21	0.13	-1.67	0.10	-0.46	0.04
Multicultural Experience × Writing Topic	0.03	0.11	0.31	0.76	-0.18	0.25
**Indirect effects**						
High Multicultural Experience (+1 SD)	-0.09	0.04			-0.17	-0.01
Low Multicultural Experience (-1 SD)	0.00	0.02			-0.05	0.04
Index of Mediated Moderation	-0.08	0.04			-0.16	-0.01

Table S2e. Sensitivity analyses results excluding influential observations in Experiment 2 (N = 402)

**Hayes Process Model 12**	***b***	***SE***	***t***	***p***	**95% CI**	
					**LL**	**UL**
**Writing Topic (Threat vs. Non-Threat) × Depth of Processing (Deliberative vs. Intuitive) × Multicultural Experience → Integrative Complexity**						
Writing Topic (1=Threat; -1=Non-Threat)	0.25	0.02	10.36	< 0.001	0.20	0.30
Depth of Processing (1=Deliberative; -1=Intuitive)	0.26	0.02	10.67	< 0.001	0.21	0.31
Writing Topic × Depth of Processing	0.11	0.02	4.47	< 0.001	0.06	0.16
Multicultural Experience	0.34	0.03	10.38	< 0.001	0.28	0.41
Writing Topic × Multicultural Experience	0.20	0.03	6.03	< 0.001	0.13	0.26
Depth of Processing × Multicultural Experience	0.24	0.03	7.37	< 0.001	0.18	0.31
Writing Topic × Depth of Processing × Multicultural Experience	0.18	0.03	5.33	< 0.001	0.11	0.24
**Conditional Effects**						
Deliberative Processing: $b = 0.37$, $F(1, 394) = 50.10$, $p < 0.001$						
High Multicultural Experience (+1 SD)	0.65	0.06	11.62	0.00	0.54	0.76
Low Multicultural Experience (-1 SD)	0.07	0.05	1.37	0.17	-0.03	0.18
Intuitive Processing: $b = 0.02$, $F(1, 394) = 0.15$, $p = 0.56$						
High Multicultural Experience (+1 SD)	0.16	0.04	3.67	0.00	0.07	0.25
Low Multicultural Experience (-1 SD)	0.13	0.05	2.78	0.01	0.04	0.21

Table S2e. Sensitivity analyses results excluding influential observations in Experiment 2 (N = 402) (cont.)

**Models**	***b***	***SE*boot**	**Bootstrap 95% CI**	
			**LL**	**UL**
**Multicultural Experience × Writing Topic (Threat vs. Non-Threat) × Depth of Processing (Deliberative vs. Intuitive) → Integrative Complexity → Stereotypes**				
Model 12				
DV: Asian Stereotypes				
**Indirect Effects**				
**Index of mediated moderation**	-0.05	0.02	-0.08	-0.02
High Multiculturalism (+1 SD)				
Deliberative Processing	-0.18	0.05	-0.28	-0.09
Intuitive Processing	-0.04	0.01	-0.08	-0.02
Index of conditional mediated moderation	-0.07	0.02	-0.11	-0.03
Low Multiculturalism (-1 SD)				
Deliberative Processing	-0.02	0.02	-0.06	0.01
Intuitive Processing	-0.03	0.01	-0.06	-0.01
Index of Conditional Mediated Moderation	0.01	0.01	-0.01	0.03
DV: Black Stereotypes				
**Indirect Effects**				
**Index of mediated moderation**	-0.04	0.02	-0.07	-0.01
High Multiculturalism (+1 SD)				
Deliberative Processing	-0.14	0.05	-0.24	-0.03
Intuitive Processing	-0.03	0.01	-0.06	-0.01
Index of Conditional Mediated Moderation	-0.05	0.02	-0.09	-0.01
Low Multiculturalism (-1 SD)				
Deliberative Processing	-0.02	0.01	-0.05	0.01
Intuitive Processing	-0.03	0.01	-0.05	0.00
Index of Conditional Mediated Moderation	0.01	0.01	-0.01	0.02

Note. Path coefficients are unstandardized estimates. *SE*s and *CI*s of indirect effects and IMMs were estimated with bootstrapping.

Exploratory Analyses

Additional measures not reported in main text—Experiment 1a

Support for peace

The study also included two established measures that assessed support for a peaceful resolution of the Israeli-Palestinian conflict. First, we measured *hope for peace* by asking participants to indicate their agreement with two items taken from previous research ($\alpha = 0.51$) (47). Second, we measured *support for concessions* necessary to make peace happen using seventeen items ($\alpha = 0.87$) (5, 48).

Results. We tested the mediated moderation effects on hope for peace and support for concessions. Neither the effects of the writing topic manipulation (hope for peace: $b = -0.01$, $p = 0.97$, 95% CI [−0.30, 0.29]; support for concessions: $b = -0.003$, $p = 0.98$, 95% CI [−0.22, 0.21]) nor those of multicultural experience (hope for peace: $b = 0.27$, $p = 0.16$, 95% CI [−0.11, 0.65]; support for concessions: $b = -0.03$, $p = 0.84$, 95% CI [−0.31, 0.25]) were significant. Although the interaction between the writing topic manipulation and multicultural experience was significant for hope ($b = 0.41$, $p = 0.04$, 95% CI [0.03, 0.79]), it was positive but non-significant for support for concessions ($b = 0.07$, $p = 0.63$, 95% CI [−0.21, 0.35]). The conditional indirect effects of multicultural experience via integrative complexity were not significant for either hope or support for concessions, and the indices of mediated moderation were also not significant using PROCESS Model 8 (hope for peace: $b = 0.07$, 95% CI [−0.06, 0.21]; support for concessions: $b = 0.08$, 95% CI [−0.01, 0.19]). Thus, although the writing topic × multicultural experience interaction reached significance for hope, the effects were inconsistent across peace measures and were not mediated by integrative complexity, indicating that the intervention primarily influenced intergroup bias against outgroups associated with the threat rather than general peace-related attitudes. Future studies should examine whether and how intergroup attitudes might be translated into more general attitudes toward reconciliation.

Endorsement of Russian and Ethiopian stereotypes

The attitudes toward Palestinian people appeared as part of a larger questionnaire that also included measures for stereotypes of Jewish Israeli minority groups (Russian people and Ethiopian people). This allows us to explore whether the manipulation effect would be generalizable beyond the focal groups associated with the ongoing threat. We did not have specific predictions about whether there would be spillover effects on other minority outgroups that were not implicated in the focal threat. The participants rated their agreement on six stereotypes of Russian ($\alpha = 0.72$) and six stereotypes toward Ethiopian ($\alpha = 0.67$; (49).

Results. The effects of the writing topic manipulation (threat vs. non-threat) (Russians: $b = 0.01$, $p = 0.91$, 95% CI [-0.16, 0.18]; Ethiopians: $b = 0.03$, $p = 0.73$, 95% CI [−0.13, 0.18]), those of multicultural experience (Russians: $b = 0.07$, $p = 0.52$, 95% CI [−0.15, 0.29]; Ethiopians: $b = -0.03$, $p = 0.73$, 95% CI [−0.24, 0.16]), and their interactions were all non-significant (Russians: $b = -0.17$, $p = 0.13$, 95% CI [−0.39, 0.05]; Ethiopians: $b = -0.15$, $p = 0.13$, 95% CI [−0.35, 0.05]).

The mediated moderation test was not significant (for endorsement of Russian stereotypes, $b = -0.06$, 95% CI [−0.14, 0.003]; for endorsement of Ethiopian stereotypes, $b = -0.04$, 95% CI [−0.11, 0.02]). However, among participants high (+1 SD) in multicultural experience ($b = -0.10$, 95% CI [−0.23, -0.01]), the significant indirect effect emerged for Russian stereotypes, indicating that individuals predisposed toward complex processing occasionally generalized increases in integrative complexity beyond the outgroups associated with the focal threat. Nevertheless, the broader pattern of results shows that deliberative processing of ongoing threat most consistently reduced bias toward outgroups associated with the threatening context (i.e., Palestinians), rather than toward unrelated minority groups. This suggests that deliberative processing of ongoing threat primarily counteracts the threat-induced narrowing of cognition around the source of the threat.

Additional measures not reported in main text—Experiment 1b

All measures are reported in the main text.

Additional measures not reported in the main text—Experiment 2

Attitudes toward republicans and democrats

We also measured participants' attitudes toward political groups. Specifically, we asked participants to indicate if they were democrat, republican or other. Those who were partisan were asked to rate the opposing group on (democrats' attitudes toward republicans: $\alpha = 0.90$; republicans' attitudes toward democrats: $\alpha = 0.88$). These measures are exploratory and were included to examine whether the effects extended beyond threat-associated intergroup contexts.

Results. We tested moderated mediation effects on democrats' attitudes toward republicans and republicans' attitudes toward democrats. Only participants who indicated their political orientation were included in this analysis. For democrats' attitudes toward republicans ($N = 196$), none of the interaction effects in the three-way interaction model between writing topic, depth of processing, and multicultural experience were significant (all ps ≥ 0.50). All conditional indirect effects were also not significant (i.e., all 95% CIs included zero). The index of mediated moderation was not significant ($b = 0.02$, 95% CI [−0.02, 0.08]).

For republicans' attitudes toward democrats ($N = 76$), none of the interaction effects in the three-way interaction model between writing topic, depth of processing, and multicultural experience were significant (ps ≥ 0.24). All conditional indirect effects were not significant (i.e., 95% CIs included zero). The index of mediated moderation was also not significant ($b = 0.06$, 95% CI [−0.07, 0.19]).

Taken together, these exploratory analyses reveal that the effects of the intervention and its interaction with multicultural experience did not meaningfully extend to political outgroups. This pattern suggests that the effects of the CTR Model might be more specific to outgroups that are more closely associated with the perceived threat, where defensive heuristic responding is

most strongly activated. In such contexts, deliberative processing is most likely to enable integrative complexity and thereby reduce bias, provided that individuals possess the cognitive readiness to translate deliberation into integratively complex reasoning.

Table S3. Survey Questions and Item Order

Table S3a. Survey Questions and Item Order, Experiment 1a

Variable name	Description
Essays	This is the open-text question for respondents to write down their essays.
Essay_difficult	This single-item measure refers to the extent of the difficulty for writing the essay.
Essay_effort	This single-item measure refers to the extent of the effort spent for writing the essay.
Essay_liketask	This single-item measure refers to the extent participants like the essay.
Essay_tiring	This single-item measure refers to the extent participants feel tiring for writing the essay.
feel1	How do you feel right now? - happy
feel2	How do you feel right now? - excited
feel3	How do you feel right now? - joyful
feel4	How do you feel right now? - proud
feel5	How do you feel right now? - afraid
feel6	How do you feel right now? - nervous
feel7	How do you feel right now? - scared
feel8	How do you feel right now? - tense
mc1	Length of time lived outside Israel out of age
mc2	Level of exposure to non-Israeli mainstream culture
mc3	The number of unique foreign languages spoken out of the total languages spoken provided
mc4	Father born outside
mc5	Mother born outside
mc6	The number of unique foreign cuisines out of the total cuisines provided
mc7	The number of unique foreign nationality of friends out of the total friends provided
mc8	The number of unique foreign musicians out of total musicians provided
p_att1	"All Palestinians are evil by nature" (Halperin et al., 2011)
p_att2	"All Palestinians really want is to destroy Israel" (Halperin et al., 2011)
p_att3	"Palestinians should never be trusted" (Halperin et al., 2011)
p_stere1	"Palestinians are generally... - violent" (Bar-Tal & Teichman, 2005)
p_stere2	"Palestinians are generally... - lazy" (Bar-Tal & Teichman, 2005)
p_stere3	"Palestinians are generally... - uneducated" (Bar-Tal & Teichman, 2005)

p_stere4	"Palestinians are generally... - disrespected life"<br>(Bar-Tal & Teichman, 2005)
p_stere5	"Palestinians are generally... - Untrustworthy"<br>(Bar-Tal & Teichman, 2005)
p_stere6	"Palestinians are generally... - manipulative"<br>(Bar-Tal & Teichman, 2005)
age	What is your age?
sex_male	What is your gender?
BF_1	Extrovert, enthusiastic<br>(Gosling et al., 2003)
BF_2	Critical, and sarcastic<br>(Gosling et al., 2003)
BF_3	Reliable, self-disciplined<br>(Gosling et al., 2003)
BF_4	Anxious, easily disappointed<br>(Gosling et al., 2003)
BF_5	Open to new experiences, complex<br>(Gosling et al., 2003)
BF_6	Restrained, quiet<br>(Gosling et al., 2003)
BF_7	Shows sympathy, warm<br>(Gosling et al., 2003)
BF_8	Disorganized, careless<br>(Gosling et al., 2003)
BF_9	Calm, emotionally stable<br>(Gosling et al., 2003)
BF_10	Conventional, not creative<br>(Gosling et al., 2003)
Politics_ForeignPolicy	Use one of the following numbers to indicate your political views in the accompanying categories. - Foreign Police<br>(Ben-Porat, 2008)
Politics_Economics	Use one of the following numbers to indicate your political views in the accompanying categories. - Economic Issues<br>(Ben-Porat, 2008)
Politics_SocialPolicy	Use one of the following numbers to indicate your political views in the accompanying categories. - Social Policy Issues<br>(Ben-Porat, 2008)
Religion_Important	Importance of religion
peace_1	I do not expect ever to achieve peace with the Palestinian people.<br>(Cohen-Chen et al., 2014)
peace_2	There is no use in really trying to end the conflict because it probably will not happen.<br>(Cohen-Chen et al., 2014)

concess_1	conduct diplomatic negotiations;<br>(Halperin et al., 2011)
concess_2	to reach an agreement of two states for two peoples<br>(Halperin et al., 2011)
concess_3	return all the settlements<br>(Halperin et al., 2011)
concess_4	unilaterally recognize Abu Mazen and the Palestinian state<br>(Halperin et al., 2011)
concess_5	return to the 1967 borders<br>(Halperin et al., 2011)
concess_6	to enter Gaza underground<br>(Halperin et al., 2011)
concess_7	recapture Gaza<br>(Halperin et al., 2011)
concess_8	to topple Hamas<br>(Halperin et al., 2011)
concess_9	recapture the West Bank<br>(Halperin et al., 2011)
concess_10	"Support for joint sovereignty over the holy places in Jerusalem";<br>(Maoz & McCauley, 2005)
concess_11	"Financial compensation for the Palestinian refugees and recognition in principle of their right to return to Israel, but this will not include the actual right to the return of the refugees";<br>(Maoz & McCauley, 2005)
concess_12	"Support for taking more conciliatory initiatives on the Israeli side";<br>(Maoz & McCauley, 2005)
concess_13	"More serious consideration of new Palestinian initiatives regarding the resolution of the conflict";<br>(Maoz & McCauley, 2005)
concess_14	"Approval of the opening of a port and an airport in Gaza";<br>(Maoz & McCauley, 2005)
concess_15	"Various concessions regarding Jerusalem";<br>(Maoz & McCauley, 2005)
concess_16	"The establishment of an independent and sovereign State of Palestine in the West Bank and the Gaza Strip, including Arab East Jerusalem and the part of the Dead Sea bordering the Palestinian coast and the territorial waters of the Gaza Strip.";<br>(Maoz & McCauley, 2005)
concess_17	"Free movement of people and goods";<br>(Maoz & McCauley, 2005)
E_stere1	"Ethiopians are generally... - primitive";<br>(Shabtai, 2001)

E_stere2	"Ethiopians are generally... - uneducated;<br>(Shabtai, 2001)
E_stere3	"Ethiopians are generally... - quiet";<br>(Shabtai, 2001)
E_stere4	"Ethiopians are generally... - unclean";<br>(Shabtai, 2001)
E_stere5	"Ethiopians are generally... - respectful to others";<br>(Shabtai, 2001)
E_stere6	"Ethiopians are generally... - cooperative";<br>(Shabtai, 2001)
R_stere1	Russians are generally... - scientifically oriented
R_stere2	Russians are generally... - condescending
R_stere3	Russians are generally... - lovers of culture
R_stere4	Russians are generally... - intelligent
R_stere5	Russians are generally... - drinkers
R_stere6	Russians are generally... - judgmental

Note. Survey questions are listed in the order they appeared to participants.

Table S3b. Survey Questions and Item Order, Experiment 1b

Variable name	Description
Essays	This is the open-text question for respondents to write down their essays.
Essay_difficult	This single-item measure refers to the extent of the difficulty for writing the essay.
Essay_effort	This single-item measure refers to the extent of the effort spent for writing the essay.
Essay_liketask	This single-item measure refers to the extent participants like the essay.
Essay_tiring	This single-item measure refers to the extent participants feel tiring for writing the essay.
feel1	How do you feel right now? - happy
feel2	How do you feel right now? - excited
feel3	How do you feel right now? - joyful
feel4	How do you feel right now? - proud
feel5	How do you feel right now? - afraid
feel6	How do you feel right now? - nervous
feel7	How do you feel right now? - scared
feel8	How do you feel right now? - tense
mc1	length of time lived outside Israel out of age
mc2	Level of exposure to non-Israeli mainstream culture divided by 10.
mc3	The number of unique foreign languages spoken out of the total languages spoken provided
mc4	The number of unique foreign cuisines out of the total cuisines provided
mc5	The number of unique foreign nationality of friends out of the total friends provided
mc6	The number of unique foreign musicians out of total musicians provided
ML_HK_1	Mainlanders in Hong Kong, just as other foreigners, are 'outsiders' (Hong et al., 2003, 2004; Tadmor et al., 2018)
ML_HK_2	Mainlanders generally have poor hygiene awareness, are lacking in public morals, and greedy (Hong et al., 2003, 2004; Tadmor et al., 2018)
ML_HK_3	Mainlanders are generally hard working, thrifty and have ambition (Hong et al., 2003, 2004; Tadmor et al., 2018)
ML_HK_4	Mainlanders have a lot of negative impacts on Hong Kong (Hong et al., 2003, 2004; Tadmor et al., 2018)
ML_HK_5	To be honest, my impression of Mainlanders is not that good (Hong et al., 2003, 2004; Tadmor et al., 2018)
ML_HK_6	Overall, I believe there are no differences between Mainlanders and Hong Kong people (Hong et al., 2003, 2004; Tadmor et al., 2018)
ML_HK_7	I feel irritable to any people or things involving Mainland China (Hong et al., 2003, 2004; Tadmor et al., 2018)
ML_HK_8	Unless it is necessary, I will not take the initiative to get in contact with people from Mainland China (Hong et al., 2003, 2004; Tadmor et al., 2018)
ML_HK_9	Many merits of the Mainland people are worth learning by Hong Kong people (Hong et al., 2003, 2004; Tadmor et al., 2018)

ML_HK_10	I feel uncomfortable to get along with the Mainlanders<br>(Hong et al., 2003, 2004; Tadmor et al., 2018)
approach1	Accept people and things in Mainland China<br>(Hong et al., 2003, 2004; Tadmor et al., 2018)
approach2	Avoid people and things in Mainland China<br>(Hong et al., 2003, 2004; Tadmor et al., 2018)
approach3	Establish good relations with people in Mainland China<br>(Hong et al., 2003, 2004; Tadmor et al., 2018)
approach4	Keep distance from or alienate people in Mainland China<br>(Hong et al., 2003, 2004; Tadmor et al., 2018)
steremainl_1	Mainlanders are in general … - impolite<br>(Hong et al., 2003, 2004; Tadmor et al., 2018)
steremainl_2	Mainlanders are in general … - hard working<br>(Hong et al., 2003, 2004; Tadmor et al., 2018)
steremainl_3	Mainlanders are in general … - unethical<br>(Hong et al., 2003, 2004; Tadmor et al., 2018)
steremainl_4	Mainlanders are in general … - competitive<br>(Hong et al., 2003, 2004; Tadmor et al., 2018)
steremainl_5	Mainlanders are in general … - uncivilized<br>(Hong et al., 2003, 2004; Tadmor et al., 2018)
steremainl_6	Mainlanders are in general … - smart<br>(Hong et al., 2003, 2004; Tadmor et al., 2018)
age	What is your age?
sex_male	What is your gender?
BF_1	Extrovert, enthusiastic<br>(Gosling et al., 2003)
BF_2	Critical, and sarcastic<br>(Gosling et al., 2003)
BF_3	Reliable, self-disciplined<br>(Gosling et al., 2003)
BF_4	Anxious, easily disappointed<br>(Gosling et al., 2003)
BF_5	Open to new experiences, complex<br>(Gosling et al., 2003)
BF_6	Restrained, quiet<br>(Gosling et al., 2003)
BF_7	Shows sympathy, warm<br>(Gosling et al., 2003)
BF_8	Disorganized, careless<br>(Gosling et al., 2003)

BF_9	Calm, emotionally stable<br>(Gosling et al., 2003)
BF_10	Conventional, not creative<br>(Gosling et al., 2003)
Politics_ForeignPolicy	Use one of the following numbers to indicate your political views in the accompanying categories. - Foreign Police<br>(Ben-Porat, 2008)
Politics_Economics	Use one of the following numbers to indicate your political views in the accompanying categories. - Economic Issues<br>(Ben-Porat, 2008)
Politics_SocialPolicy	Use one of the following numbers to indicate your political views in the accompanying categories. - Social Policy Issues<br>(Ben-Porat, 2008)

Note. Survey questions are listed in the order they appeared to participants.

Table S3c. Survey Questions and Item Order, Experiment 2

Variable name	Description
Essays	This is the open-text question for respondents to write down their essays.
mc1	Length of time lived outside Israel out of age
mc2	Level of exposure to non-Israeli mainstream culture divided by 10.
mc3	The number of unique foreign languages spoken out of the total languages spoken provided
mc4	Father born outside
mc5	Mother born outside
mc6	The number of unique foreign cuisines out of the total cuisines provided
mc7	The number of unique foreign nationality of friends out of the total friends provided
mc8	The number of unique foreign musicians out of total musicians provided
sterasian_1	Asians / Asian-Americans are in general … - ... unsociable. (Ho & Jackson, 2001)
sterasian_2	Asians / Asian-Americans are in general … - ... shy. (Ho & Jackson, 2001)
sterasian_3	Asians / Asian-Americans are in general … - ... superstitious. (Ho & Jackson, 2001)
sterasian_4	Asians / Asian-Americans are in general … - ... humorless. (Ho & Jackson, 2001)
sterasian_5	Asians / Asian-Americans are in general … - ... non-athletic. (Ho & Jackson, 2001)
sterasian_6	Asians / Asian-Americans are in general … - ... obedient. (Ho & Jackson, 2001)
sterblack_1	Blacks / African-Americans are in general … - ... uneducated. (Levy et al., 1998)
sterblack_2	Blacks / African-Americans are in general … - ... violent. (Levy et al., 1998)
sterblack_3	Blacks / African-Americans are in general … - ... irresponsible. (Levy et al., 1998)
sterblack_4	Blacks / African-Americans are in general … - ... sexual. (Levy et al., 1998)
sterblack_5	Blacks / African-Americans are in general … - ... lazy. (Levy et al., 1998)
sterblack_6	Blacks / African-Americans are in general … - ... undisciplined. (Levy et al., 1998)
politics_party	Political orientation/affiliation
negRep_1	describing - Republicans: good - bad
negRep_2	describing - Republicans: industrious - lazy
negRep_3	describing - Republicans: smart - stupid

negRep_4	describing - Republicans: clean - dirty
negRep_5	describing - Republicans: beautiful - ugly
negRep_6	describing - Republicans: tempered - violent
negDem_1	describing Democrats: good - bad
negDem_2	describing Democrats: industrious - lazy
negDem_3	describing Democrats: smart - stupid
negDem_4	describing Democrats: clean - dirty
negDem_5	describing Democrats: beautiful - ugly
negDem_6	describing Democrats: tempered - violent
age	What is your age?
sex_male	What is your gender?

Note. Survey questions are listed in the order they appeared to participants.

References

1. B. L. Burke, A. Martens, E. H. Faucher, Two decades of terror management theory: A meta-analysis of mortality salience research. *Personality and Social Psychology Review* **14**, 155–195 (2010).
2. J. W. Pennebaker, Writing about emotional experiences as a therapeutic process. *Psychol. Sci.* **8**, 162–166 (1997).
3. A. K. Leung, W. W. Maddux, A. D. Galinsky, C. Chiu, Multicultural experience enhances creativity: The when and how. *American Psychologist* **63**, 169–181 (2008).
4. A. K. Leung, C. Chiu, Multicultural experience, idea receptiveness, and creativity. *J. Cross. Cult. Psychol.* **41**, 723–741 (2010).
5. E. Halperin, A. G. Russell, K. H. Trzesniewski, J. J. Gross, C. S. Dweck, Promoting the Middle East peace process by changing beliefs about group malleability. *Science (1979).* **333**, 1767–1769 (2011).
6. D. Bar-Tal, Y. Teichman, *Stereotypes and prejudice in conflict: Representations of Arabs in Israeli Jewish society* (Cambridge University Press, 2005).
7. K. Ng, Don't blame new migrants from mainland China for Hong Kong's housing and health care woes, rights groups say while calling for race hate law. *South China Morning Post* (2019). Available at: https://www.scmp.com/news/hong-kong/politics/article/2184889/dont-blame-new-migrants-mainland-china-hong-kongs-housing [Accessed 26 September 2025].
8. W.-K. B. Wong, "Discrimination against the mainland Chinese and Hong Kong's defense of local identity" in *China's New 21st Century Realities: Social Equity in a Time of Change*, R. G. Johnson, Ed. (Peter Lang New York, 2015), pp. 23–37.
9. C. T. Tadmor, Y. Hong, M. M. Chao, A. Cohen, The tolerance benefits of multicultural experiences depend on the perception of available mental resources. *J. Pers. Soc. Psychol.* **115**, 398–426 (2018).
10. Y. Hong, *et al.*, How are social identities linked to self-conception and intergroup orientation? The moderating effect of implicit theories. *J. Pers. Soc. Psychol.* **85**, 1147–1160 (2003).
11. Y.-Y. Hong, *et al.*, Predicting intergroup bias: The interactive effects of implicit theory and social identity. *Pers. Soc. Psychol. Bull.* **30**, 1035–1047 (2004).
12. G. Baker-Brown, *et al.*, "The conceptual/integrative complexity scoring manual" in *Motivation and Personality: Handbook of Thematic Content Analysis*, C. P. Smith, Ed. (Cambridge University Press., 1992), pp. 401–418.
13. A. F. Hayes, *Introduction to mediation, moderation, and conditional process analysis: A regression-based approach (2nd ed.)* (Guilford publications, 2018).
14. J. W. Pennebaker, Putting stress into words: Health, linguistic, and therapeutic implications. *Behaviour Research and Therapy* **31**, 539–548 (1993).
15. W. W. Maddux, A. D. Galinsky, C. T. Tadmor, Be a better manager: Live abroad. *Harv. Bus. Rev.* **88**, 24 (2010).
16. C. T. Tadmor, A. D. Galinsky, W. W. Maddux, Getting the most out of living abroad: biculturalism and integrative complexity as key drivers of creative and professional success. *J. Pers. Soc. Psychol.* **103**, 520–542 (2012).
17. J. T. Crawford, M. J. Brandt, Who is prejudiced, and toward whom? The big five traits and generalized prejudice. *Pers. Soc. Psychol. Bull.* **45**, 1455–1467 (2019).
18. B. Ekehammar, N. Akrami, T. Araya, Gender differences in implicit prejudice. *Pers. Individ. Dif.* **34**, 1509–1523 (2003).

19. S. D. Gosling, P. J. Rentfrow, W. B. Swann Jr, A very brief measure of the Big-Five personality domains. *J. Res. Pers.* **37**, 504–528 (2003).
20. G. Ben-Porat, "Political economy: liberalization and globalization" in *Israel since 1980*, (Cambridge University Press, 2008), pp. 91–116.
21. M. Kremnitzer, A. Fuchs, "The non-separation of religion and state in Israel: does it support the racism and nationalism wave" in *Handbook of Israel: Major Debates Edited by Eliezer Ben-Rafael, Julius H. Schoeps, Yitzhak Sternberg and Olaf Glöckner.*, (De Gruyter Oldenbourg, Berlin/Boston, 2016), pp. 181–196.
22. B. Bago, W. De Neys, The intuitive greater good: Testing the corrective dual process model of moral cognition. *J. Exp. Psychol. Gen.* **148**, 1782–1801 (2019).
23. B. Bago, W. De Neys, Fast logic?: Examining the time course assumption of dual process theory. *Cognition* **158**, 90–109 (2017).
24. B. Bago, D. G. Rand, G. Pennycook, Does deliberation decrease belief in conspiracies? *J. Exp. Soc. Psychol.* **103**, 104395 (2022).
25. A. Miyake, N. P. Friedman, D. A. Rettinger, P. Shah, M. Hegarty, How are visuospatial working memory, executive functioning, and spatial abilities related? A latent-variable analysis. *J. Exp. Psychol. Gen.* **130**, 621–640 (2001).
26. W. De Neys, W. Schaeken, When people are more logical under cognitive load: Dual task impact on scalar implicature. *Exp. Psychol.* **54**, 128–133 (2007).
27. S. Franssens, W. De Neys, The effortless nature of conflict detection during thinking. *Think. Reason.* **15**, 105–128 (2009).
28. C. T. Tadmor, Y. Hong, M. M. Chao, F. Wiruchnipawan, W. Wang, Multicultural experiences reduce intergroup bias through epistemic unfreezing. *J. Pers. Soc. Psychol.* **103**, 750–772 (2012).
29. C. T. Tadmor, P. Satterstrom, S. Jang, J. T. Polzer, Beyond individual creativity: The superadditive benefits of multicultural experience for collective creativity in culturally diverse teams. *J. Cross. Cult. Psychol.* **43**, 384–392 (2012).
30. Y. Liu, B. K. Finch, S. G. Brenneke, K. Thomas, P. D. Le, Perceived discrimination and mental distress amid the COVID-19 pandemic: evidence from the understanding America study. *Am. J. Prev. Med.* **59**, 481–492 (2020).
31. N. G. Ruiz, J. M. Horowitz, C. Tamir, Many Black and Asian Americans say they have experienced discrimination amid the COVID-19 outbreak. *Pew Research Center* (2020). Available at: https://www.pewresearch.org/social-trends/2020/07/01/many-black-and-asian-americans-say-they-have-experienced-discrimination-amid-the-covid-19-outbreak/ [Accessed 26 September 2025].
32. J. T. Huang, M. Krupenkin, D. Rothschild, J. Lee Cunningham, The cost of anti-Asian racism during the COVID-19 pandemic. *Nat. Hum. Behav.* **7**, 682–695 (2023).
33. A. Makhanova, The behavioral immune system and intergroup bias: Evidence for Asian-specific bias at the onset of the COVID-19 Pandemic. *Evol. Psychol. Sci.* **8**, 333–342 (2022).
34. K. Mackey, *et al.*, Racial and ethnic disparities in COVID-19–related infections, hospitalizations, and deaths: a systematic review. *Ann. Intern. Med.* **174**, 362–373 (2021).
35. E. Ash, K. Schulenberg, M. Wilson, S. Mikkilineni, Framing risk and responsibility: Newspaper coverage of COVID-19 racial disparities. *Newsp. Res. J.* **44**, 174–189 (2023).

36. J. Gordils, A. J. Elliot, S. Toprakkiran, J. P. Jamieson, The effects of COVID-19 on perceived intergroup competition and negative intergroup outcomes. *J. Soc. Psychol.* **161**, 419–434 (2021).
37. T. M. Mandalaywala, G. Gonzalez, L. R. Tropp, Early perceptions of COVID-19 intensity and anti-Asian prejudice among White Americans. *Group Processes & Intergroup Relations* **26**, 48–70 (2023).
38. D. Qi, J. C. Garand, Americans' Attitudes Towards Asians in the Trump COVID Era. *American Politics Research* **53**, 425–439 (2025).
39. C. Ho, J. W. Jackson, Attitude toward asian americans: Theory and measurement. *J. Appl. Soc. Psychol.* **31**, 1553–1581 (2001).
40. S. R. Levy, S. J. Stroessner, C. S. Dweck, Stereotype formation and endorsement: The role of implicit theories. *J. Pers. Soc. Psychol.* **74**, 1421–1436 (1998).
41. H. Aguinis, R. K. Gottfredson, H. Joo, Best-practice recommendations for defining, identifying, and handling outliers. *Organ. Res. Methods* **16**, 270–301 (2013).
42. R. M. O'brien, A caution regarding rules of thumb for variance inflation factors. *Qual. Quant.* **41**, 673–690 (2007).
43. A. Field, *Discovering statistics using IBM SPSS statistics* (Sage publications limited, 2024).
44. J. Cohen, P. Cohen, S. G. West, L. S. Aiken, *Applied multiple regression/correlation analysis for the behavioral sciences* (Routledge, 2013).
45. R. D. Cook, S. Weisberg, Residuals and influence in regression. (1982).
46. D. C. Montgomery, E. A. Peck, G. G. Vining, *Introduction to linear regression analysis* (John Wiley & Sons, 2021).
47. S. Cohen-Chen, E. Halperin, R. J. Crisp, J. J. Gross, Hope in the Middle East: Malleability beliefs, hope, and the willingness to compromise for peace. *Soc. Psychol. Personal. Sci.* **5**, 67–75 (2014).
48. I. Maoz, C. McCauley, Psychological correlates of support for compromise: A polling study of Jewish-Israeli attitudes toward solutions to the Israeli-Palestinian conflict. *Polit. Psychol.* **26**, 791–808 (2005).
49. M. Shabtai, Living with a threatening identity: Life experiences with a difference in skin color among Ethiopian youngsters and adolescents in Israel. *Megamot* **41**, 72–97 (2001).
